# Human somatic cells subjected to genetic induction with six germ line-related factors display meiotic germ cell-like features

**DOI:** 10.1038/srep24956

**Published:** 2016-04-26

**Authors:** Jose V. Medrano, Ana M. Martínez-Arroyo, Jose M. Míguez, Inmaculada Moreno, Sebastián Martínez, Alicia Quiñonero, Patricia Díaz-Gimeno, Ana I. Marqués-Marí, Antonio Pellicer, Jose Remohí, Carlos Simón

**Affiliations:** 1 Fundación Instituto Valenciano de Infertilidad (FIVI), INCLIVA, Department of Pediatrics, Obstetrics and Gynecology, Valencia University, Valencia, 46015, Spain; 2 Fundación Instituto de Investigación Sanitaria La Fe, Valencia, 46026, Spain; 3 Igenomix S.L., Paterna, 46980, Spain; 4 Valencia Node of the Spanish Stem Cell Bank, Prince Felipe Research Centre (CIPF) Valencia, 46012, Spain; 5 Department of Obstetrics and Gynecology, Stanford University, Stanford, CA 94305, USA

## Abstract

The *in vitro* derivation of human germ cells has attracted interest in the last years, but their direct conversion from human somatic cells has not yet been reported. Here we tested the ability of human male somatic cells to directly convert into a meiotic germ cell-like phenotype by inducing them with a combination of selected key germ cell developmental factors. We started with a pool of 12 candidates that were reduced to 6, demonstrating that ectopic expression of the germ line-related genes *PRDM1, PRDM14, LIN28A, DAZL, VASA* and *SYCP3* induced direct conversion of somatic cells (hFSK (46, XY), and hMSC (46, XY)) into a germ cell-like phenotype *in vitro*. Induced germ cell-like cells showed a marked switch in their transcriptomic profile and expressed several post-meiotic germ line related markers, showed meiotic progression, evidence of epigenetic reprogramming, and approximately 1% were able to complete meiosis as demonstrated by their haploid status and the expression of several post-meiotic markers. Furthermore, xenotransplantation assays demonstrated that a subset of induced cells properly colonize the spermatogonial niche. Knowledge obtained from this work can be used to create *in vitro* models to study gamete-related diseases in humans.

In USA, an estimated 220,000 men and 290,000 women aged 20–44 lack gametes to produce their own genetic offspring^1^. Although donation of gametes results in high pregnancy rates, there are ethical, legal and personal concerns associated with this technique. Thus, there is an increasing interest in the search for alternatives to generate autologous germ cells *in vitro*.

In mice, the germ line is originated from a founder population of mesodermal cells located in the proximal epiblast that escapes the somatic fate at the moment of gastrulation in response to bone morphogenetic proteins 4, 7, and 8b (BMP4, BMP7 and BMP8b) from the adjacent extra-embryonic endoderm, constituting the primordial germ cells (PGCs)^2^,^3^. After PGC specification, murine germ cells go through several developmental stages that are tightly regulated both at the transcriptional level by transcription factors, but also at the translational level by RNA-binding proteins. Moreover, during their development germ cells suffer a complete germline-specific epigenetic remodeling that is essential to coordinate its development^4^.

Based on our knowledge of the murine germ line development, here we present a model for direct germ cell-like conversion from human somatic cells *in vitro* by genetic induction of selected key germ cell factors. Although reports of germ line differentiation from human pluripotent stem cells already exist^5^,^6^,^7^,^8^,^9^,^10^,^11^, this work can be considered the first evidence of fate directed conversion from a somatic cell origin into a germ cell-like phenotype by genetic induction.

## Results

### Induction of human foreskin fibroblasts (hFSKs) and human mesenchymal stem cells (hMSCs) using germ line factors triggers the formation of germ cell-like cells

Initially, we identified a pool of 12 candidate genes (i12F), with unequivocal contribution in the mammalian germ line determination, migration and meiotic progression in the mouse model: *PRDM1*^2^,^12^, *PRDM14*^13^, *LIN28A*^2^, *NANOG*^14^, *NANOS3*^15^, *DAZ2, BOLL, DAZL*^16^, *VASA* (also known as *DDX4*)^17^,^18^,^19^, *STRA8*^20^, *DMC1*^21^ and *SYCP3*^22^. Next, we cloned their Consensus Coding Sequences (CCDSs) into a lentiviral expression backbone with a CMV promoter and a green fluorescent protein (GFP) reporter (pLenti7.3/V5-DEST, Life Technologies), and used lentiviral particles to transduce them into human foreskin fibroblasts (hFSK) (46, XY) (Supplementary Figure S1C).

Initially, fibroblasts transduced with i12F were cultured in standard medium comprising DMEM/F12 with 10% FBS. Starting between days 3–7 post-transduction, we observed morphological changes in the fibroblast-like cultures with the appearance of round single cells that formed clumps of compacted small cells. However, after a few days, these clumps disappeared, suggesting that the culture conditions were not sufficient for their survival (Supplementary Figure S3A). Therefore, we based in previous reports on *in vitro* derivation of Spermatogonial Stem Cells (SSCs)^23^,^24^,^25^ to design a Germ Cell Medium (GC-M) enriched with several growth factors to promote the survival of the putative germ cells resulting from genetic induction (see Methods section for further details). Replacing stardard medium by GC-M at 24h post-transduction resulted in an increase of cell clumps formation (Supplementary Figure S3A). Thus, GC-M was employed for culturing both MOCK and induced cells *in vitro* in following experiments.

Transduced fibroblasts showed a clear up-regulation of all 12 induced factors during the first week post-transduction, with a marked decrease during the second and third week for most of the transgenes, probably due to the silencing of the CMV promoter driving its expression (Supplementary Figure S1A). However, further expression analysis at day 14 post-transduction indicated that transgenes continued their expression still at moderate levels (Supplementary Figure S1B). This observation was corroborated by a detectable GFP signal that did not disappear along time (Supplementary Figure S2A).

**Figure 1:**
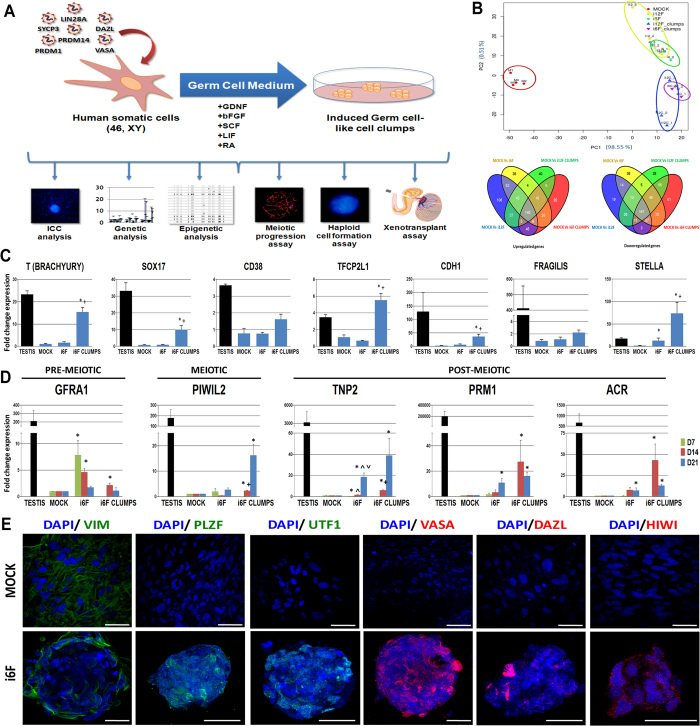
Characterization of induced fibroblasts (hFSKs). (**A)** Schematic diagram of the experimental setup of the study. **(B)** Principal Component Analyses and Venn diagrams of up- and down-regulated genes when compared MOCK, i12F and i6F- induced hFSKs all together (n = 5). **(C)** RT-qPCR expression analysis of human PGC markers over i6F induced hFSK cells. **(D)** RT-qPCR expression analysis of the germ line markers *GFRA1, PIWIL2, TNP2, PRM1*, and *ACR* over i6F induced hFSK cells at 7 (D7), 14 (14D) and 21 (21D) days post-transduction (n = 8). Human testis cDNA physiological expression fold change relative to MOCK samples is also shown as a control. **(E)** Illustrative pictures of immunofluorescencent stainings for VIM, PLZF, UTF1, VASA, DAZL and HIWI over MOCK and i6F clumps from hFSK cells. Data is presented as normalized fold change mean +/− SEM. (*) represent significant differences (p < 0.05) with MOCK controls; (+) represents significant differences (p < 0.05) between i12F/i6F conditions and their respective clumps; (∧) represent significant differences (p < 0.05) with day 7 expression within sample groups; (∨) represent significant differences (p < 0.05) with day 14 expression within sample groups. Scale bar represents a distance of 50 μm.

Initial characterization of i12F transduced hFSK cells indicated a significant up-regulation of the epithelial marker E-Cadherin (CDH1) and the PGC germ cell marker STELLA specifically in the clumps two weeks post-transduction. Although not significant, FRAGILIS, another known PGC marker, also showed a relative up-regulation in the clumps, suggesting their possible germ cell-like identity (Supplementary Figure S3B). Next, we sought to find the minimal combination of factors necessary for the phenotypic switch achieved with the i12F cocktail. For this, we screened among the different combinations of factors within i12F employing as a read out the efficiency of clump formation from hFSK cells. We individually transduced all twelve factors and selected those factors that induced the appearance of clumps. Afterwards, we designed factorial combinations of factors to achieve the maximum efficiency of clump formation by microscopic observation (Supplementary Figure S2). As a result of this screening, the most effective combination was the combined ectopic expression of: *PRDM1, PRDM14, LIN28, DAZL* and *VASA.* Additionally to these five factors, ectopic expression of SYCP3 resulted essential for achieving the meiotic-like phenotype described below (see Discussion). Thus, next experiments employed a cocktail of 6 factors comprising *PRDM1, PRDM14, LIN28, DAZL, VASA* and *SYCP3* (i6F) (Fig. 1A).

Principal components analysis (PCA) of gene expression profile 14 days after transduction clustered i12F/i6F and i12F clumps/i6F clumps in two defined groups different that MOCK controls (Fig. 1B and Supplementary Figure S3D). Moreover, i6F cells showed a switch in their genetic expression program, with the significant up-regulation of 293 genes and the down-regulation of 322 genes compared to MOCK controls. Manually isolated i6F clumps showed significant up-regulation of 442 genes (226 of them shared with i12F) and down-regulation of 402 genes (254 of them shared with i12F treatment) compared to MOCK controls. Further comparisons between i12F and i6F identified 140 significant up-regulated genes and 167 down-regulated genes shared between induced cells, compared to MOCK controls (Fig. 1B). Functional enrichment analysis of the list of differentially regulated genes in i6F indicated their implication in germ cell-related processes such as “Integrin cell surface interactions” (REACT_13552), “Cell cycle” (REACT_152), “DNA Replication” (REACT_383), “Telomere maintenance (REACT_7970), as well as several Gene Ontology biological processes related to “Positive regulation of MAP kinase activity” (GO:0043406), “ovarian follicle development” (GO:0001541), “positive regulation of tyrosine phosphorylation of STAT protein” (GO:0042531), “Retinoid acid metabolic process” (GO:0042573) and “transforming growth factor beta receptor signaling pathway” (GO:0007179), among others. Interestingly, we observed the significant down-regulation of several genes related to the mitotic cell cycle regulation, and the significant up-regulation of genes related to the TGFβ and LIF/STAT3 pathways. On the other hand, clumps showed a significant down-regulation of several genes related to chromatin stability and several somatic lineage determinant factors (Supplementary Table S1).

In a similar way that happened with i12F, i6F induced cell clumps showed significant up-regulation for the human PGC markers CDH1 and STELLA (Fig. 1C), but also for the recently described human PGC markers T (Brachyury), SOX17, and the naïve pluripotency marker TFCP2L1, indicating the presence of PGC-like cells within clumps (Fig. 1C). However, further RT-qPCR time course analysis showed a significantly increased expression of the pre-meiotic spermatogonial marker GDNF receptor type 1α (GFRA1) during the first week post-transduction that remained over the second week, and declined at the third week post-transduction. Concomitant with this, we observed a trend of the data to show up-regulation of the meiotic (PIWIL2) and post-meiotic markers Transition Protein 2 (TNP2), Protamin 1 (PRM1) and Acrosin (ACR), that resulted statistically significant from the second week post-transduction onwards in the case of TNP2, and from the third week post-transduction in the case of PRM1 and ACR (Fig. 1D). More significant differences with MOCK controls were found along time in manually isolated clumps for PIWIL2, TNP2, PRM1 and ACR, but not for GFRA1, suggesting that a more differentiated germ cell-like population might have arised and co-exist with pre-meiotic germ cell-like cells within the clumps. However, only PIWIL2 and TNP2 showed significant differences 14 days post-transduction in clumps compared with the whole i6F culture (Fig. 1D). Similar results were observed in i12F induced cultures, highlighting the similarities between 12F and i6F phenotypes (Supplementary Figure S3C). Immunocytological analysis demonstrated the expression of the germ line-related markers VASA and DAZL (both exogenously induced), HIWI, PLZF and UTF1 in all the analyzed clumps, whereas the mesenchymal filament VIMENTIN (VIM) was found to be expressed only in fibroblastic cells surrounding the clumps but not within them (Fig. 1E).

In order to test if this phenotype may also appear by using another cell source, we also transduced human mesenchymal cells derived from bone marrow (hMSCs, 46, XY) with the i12F and i6F cocktails. In contrast with our observations in hFSK induced cells, where most of the cells kept their fibroblastic morphology and some clumps raised, in hMSCs most of the cells detached from the culture plates during the first week after the lentiviral transduction and those that remained attached switched their morphology to form clumps (Supplementary Figure S5A). Interestingly, hMSCs responded with the maximal expression of post-meiotic markers during the first week post-transduction, whereas in hFSK cells it occurred at the third week, indicating a different timing in the reprogramming process between both cell types (Supplementary Figure S5D).

### Induced germ cell-like cells progress through meiosis *in vitro*

**Figure 2:**
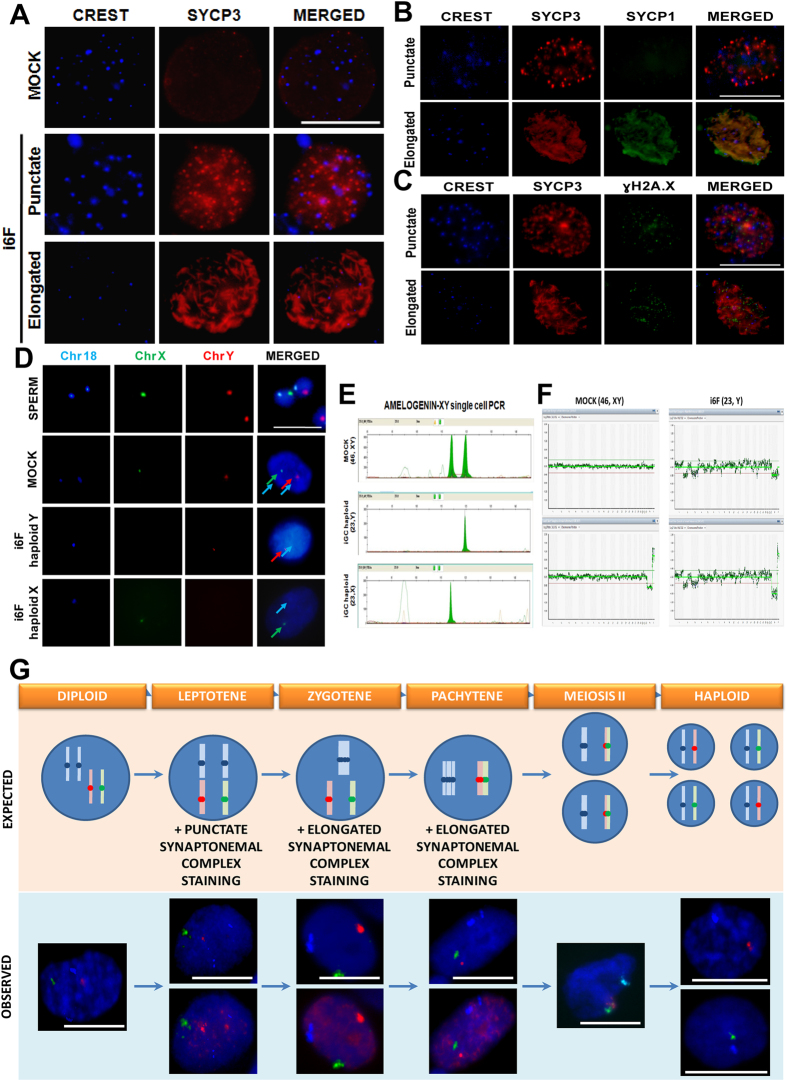
Meiotic progression analysis 14 days post-transduction of fibroblasts. (**A)** Illustrative pictures of the SYCP3 staining pattern over i6F transduced hFSK cells. **(B)** SYCP3 and SYCP1 co-localization over transduced cells indicates effective chromosomal synapsis. **(C)** SYCP3 and ɣH2A.X co-localization over transduced cells indicates putative DSB loci. **(D)** Representative FISH results for probes against chromosomes 18 (aqua), X (green) and Y (red) over 1N sorted cells. **(E)** Molecular assessment of the ploidy in single cells. PCR products of the Amelogenin gene results in a peak of 118pb for the copy in X and a 124pb peak for the copy in Y. **(F)** Illustrative aCGH results of a diploid (46, XY) cell from MOCK and a haploid (23, Y) cell from i6F, co-hybridized with male (upper panels) and female (lower panels) diploid references. **(G)** Combined SYCP3 staining and FISH analysis reveals that meiotic-like cells recapitulate all the stages of the meiosis. The upper section shows the expected pattern of centromeric probes for the chromosomes 18, X and Y over meiotic cells. The bottom section shows representative pictures of the combined SYCP3 stainning (dark red) and FISH analysis corresponding to each of the meiotic sub-stages. In diploid cells, the expected FISH pattern in (46, XY) cells is 2 aqua signals: 1 green signal: 1 red signal (2:1:1), and any SYCP3 staining. During leptotene, cells show a FISH pattern 2:1:1, with a punctate SYCP3 staining. Since zygotene is a transitional sub-stage until homologue chromosomes are totally paired, both 1:1:1 and 2:1:1 FISH patterns are possible, co-localizing with an elongated SYCP3 staining. In pachytene, totally paired homologue chromosomes show a FISH pattern 1:1:1 with overlap of X and Y signals in the bivalent structure and an elongated SYCP3 staining. After the first reductional meiotic division, the synaptonemal complex is undetectable and nuclei show a 1:1:1 FISH pattern with overlapped X and Y signals. Finally, after the second equational meiotic division, haploid cells can either show a 1:0:1 or a 1:1:0 FISH pattern. Data is presented as mean +/− SEM. (**) represent significant differences (p < 0.01) with MOCK controls. Scale bar represents a distance of 10 μm.

The expression of post-meiotic markers in induced cells prompted us to analyze their meiotic status. We assessed the localization and distribution of the axial element of the synaptonemal complex protein 3 (SYCP3) to determine the prophase I meiotic status on day 14 post-transduction. Although slightly different from the nuclear meiotic patterns previously reported in mammalian spermatocytes^26^, SYCP3 positive nuclei were considered meiotic when showed a punctate staining pattern corresponding to leptotene, or an elongated pattern corresponding to late zygotene and forward stages of the prophase I (Fig. 2A), as previously reported^7^,^8^,^9^,^11^,^27^.

Although several nuclei resulted positive for SYCP3 staining due to the exogenous expression of SYCP3, most of them showed an aberrant pattern resembling protein-like aggregations (Supplementary Figure S4C). Thus, only 1.88% of hFSK i6F cells showed a proper punctuate/elongated SYCP3 staining pattern corresponding to the prophase I of the meiosis, indicating a low efficiency of spontaneous meiotic entry of induced cells. Approximately 30.85% of meiotic-like nuclei (0.65% of the total population) had an elongated SYCP3 pattern, whereas 69.15% (1.23% of the total population) showed a punctate pattern (Supplementary Figure S4B). Similar results were obtained when cells were induced with the complete i12F cocktail (Supplementary Figure S4A,B). When hMSCs were subjected to the same genetic induction with both i12F and i6F cocktails, approximately 2.02% showed a correct positive SYCP3 staining pattern. The percentage of meiotic-like cells with elongated SYCP3 pattern in the i6F induced hMSCs was 56.93% (1.15% of the total population), whereas 43.07% (0.87% of the total population) showed a punctate staining pattern (Supplementary Figure S5C).

Chromosomal synapsis was detected by co-localization of SYCP1 and SYCP3 (Fig. 2B). Additionally, ɣH2A.X co-localization with SYCP3 indicated the appearance of SPO11-independent double strand breaks (DSBs) (Fig. 2C). However, even that we observed chromosomal synapsis between homologs and putative DSBs, we did not detect recombination events by MLH1 staining (data not shown).

In order to confirm if meiotic-like cells were able to fully complete meiosis, we next stained cells with propidium iodide (PI) and isolated the putative haploid (1N) population by fluorescence activated cell sorting (FACS) (Supplementary Figure S4D) as previously reported^7^,^8^,^9^. We were able to isolate 1–2% putative haploid cells from i6F induced cultures, a figure which correlates with the percentage of meiotic-like cells found by SYCP3 staining (Supplementary Figure S4B). When fluorescence *in situ* hybridization (FISH) with centromeric probes for the chromosomes 18, X and Y was performed (Fig. 2D), we found that 12.15% of hFSK cells within the putative 1N i6F sorted populations resulted haploid, compared to 1.07% of haploid cells within the unsorted populations (Supplementary Figure S4F). Since i6F clumps showed a significant enrichment of the expression of post-meiotic markers, we also performed FISH analysis over them after their manual isolation, resulting in an enrichment of 5.86% of haploid cells (Supplementary Figure S4F). In the case of hMSCs, we found that 4.11% of the i6F induced hMSCs resulted haploid, whereas we observed an enrichment of 20% haploid cells within the putative 1N sorted cell population (Supplementary Figure S5C). Complete FISH-based counts can be found in Supplementary Tables S2 and S3.

Haploidy of cells was validated by re-hybridization of the same nuclei with centromeric probes for the chromosomes 10, 12 and 3 (Supplementary Figure S4E). These results were also supported by an alternative molecular approach based in the detection of the AMELOGENIN gene, which has been widely used in sex determination of unknown human samples by PCR^28^. Here, we employed the 6bp length difference between both X and Y chromosome gene copies of AMELOGENIN to determine the presence/absence of sexual chromosomes in induced single cells individually isolated and used it to confirm their ploidy (Fig. 2E). Further array Comparative Genomic Hybridization (aCGH) analysis with single cells from clumps confirmed the haploidy of the cells previously analyzed by AMELOGENIN PCR (Fig. 2F).

Finally, combination of both meiotic analysis by SYCP3 staining and ploidy analysis by FISH over the same nuclei led us to identify all the intermediate meiotic stages of the prophase I from pre-leptotene to pachytene (Fig. 2G).

### i6F induction leads to the activation of the TET-mediated methylation erasure pathway

**Figure 3:**
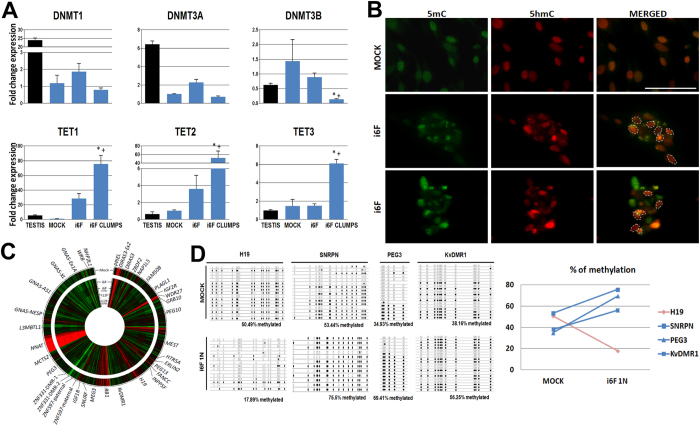
Epigenetic characterization of the *in vitro* induced fibroblasts. (**A)** RT-qPCR expression analysis of the DNA methyl-transferases DNMT1, DNMT3A and DNMT3B, and the TET-mediated de-methylases TET1, TET2 and TET3 over i6F induced fibroblasts 14 days post-transduction (n = 3). Human testis cDNA physiological expression fold change relative to MOCK samples is also shown as a control. Data is presented as normalized fold change mean +/− SEM. (*) represent significant differences (p < 0.05) with controls; (+) represents significant differences (p < 0.05) between i6F whole culture condition and i6F clumps. **(B)** Representative pictures of the co-localization of 5-methyl-Cytosine (5mC) and 5-hydroxi-methyl-Cytosine (5hmC) over MOCK cells and i6F clumps at 14 days post-transduction. Dashed lines indicate cell nuclei enriched for 5hmC (red) in MOCK control (0/15 nuclei in the picture) and in two illustrative pictures of i6F clumps (7/17 nuclei (41.1%) and 5/18 nuclei (27.7%), respectively) compared with the 5mC signal (green). Scale bar represents a distance of 10 μm. **(C)** Circular heat map presentation of the methylation for 37 annotated human imprinted loci in induced fibroblasts 14 days post-transduction (n = 3). **(D)** Bisulphite sequencing results at 14 days post-transduction at the DMR of the *H19, SNRPN, PEG3* and *KvDMR1 loci* in putative 1N sorted i6F cells (n = 3). Diagrams represent methylation status of each CpG dinucleotide on individual DNA clones. Lines represent different clones and columns are different CpG dinucleotides. Methylated CpGs are represented as filled circles and unmethylated CpGs are represented as open circles. Empty CpG sites represent the CpGs that could not be determined. In the graph, blue line indicates the methylation change in the analyzed maternally imprinted loci, whereas pink line indicates the methylation change observed for the paternally imprinted loci *H19*.

We observed an up-regulation of the Ten-eleven translocation methylcytosine dioxygenases TET1, TET2 and TET3 in i6F induced fibroblasts that was significantly higher in the clumps compared to the rest of the culture (Fig. 3A), suggesting the activation of this de-methylation pathway. This finding was supported by the detection of the marker 5-hydroxi-methyl-Cytosine (5hmC), which was previously described as a mark for the CpGs to be de-methylated by the TET pathway^29^. Interestingly, we also observed a relative enrichment of 5hmC within the clumps during the second week of culture after transduction (Fig. 3B). Slight differences in the DNA methyltransferases DNMT1, DNMT3A and DNMT3B in induced fibroblasts versus controls were observed, except a decrease in the expression of the de novo DNMT3B in i6F clumps (Fig. 3A).

Methylation arrays indicated very slight differences between MOCK controls and induced cells (Fig. 3C). Only haploid enriched 1N i6F sorted cells showed a specific decrease of methylation in the paternally imprinted *H19* DMR, whereas the maternally imprinted DMRs in *SNRPN, PEG3* and *KvDMR1* loci increased their methylation (Fig. 3D), suggesting that this population of cells enriched for putative haploid cells may have acquired a female gamete-like epigenetic profile.

### Germ cell xenotransplantation assay demonstrates that i6F induced cells are able to colonize seminiferous tubules

To test the functionality of i6F induced hFSK cells, we employed busulfan treated NUDE male mice as recipients to test the ability of MOCK and i6F transduced cells to colonize their germ cell-depleted seminiferous epithelium as previously described^30^,^31^.

Small green fluorescence protein (GFP) dots appeared in some seminiferous tubules of i6F-transplanted testis, suggesting the presence of colonies of induced cells within them (Supplementary Figure S6A). Additionally, and even that we observed focal spontaneous recovery of mouse spermatogenesis in both transplanted and not transplanted busulfan treated mice, we observed that i6F transplanted testis showed a slight increase in the normalized testis weight when compared to other conditions (Supplementary Figure S6B).

**Figure 4:**
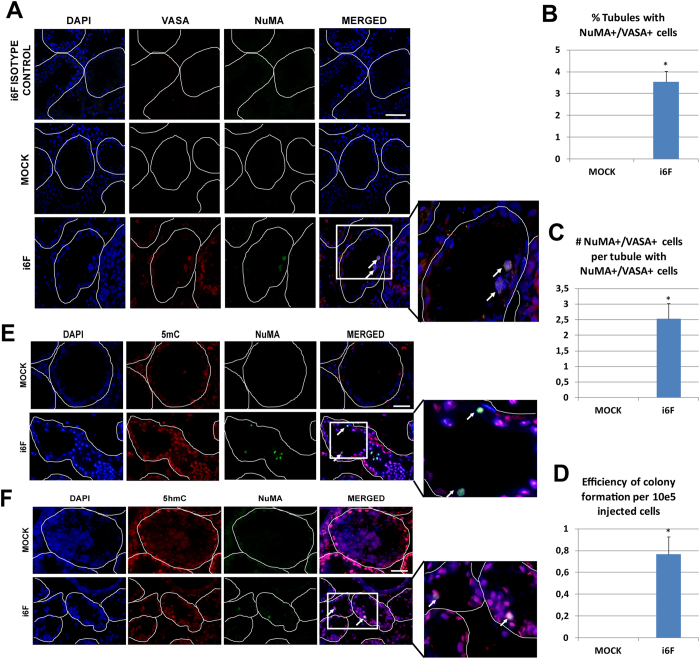
Germ cell xenotransplant results. (**A)** Illustrative pictures showing NuMA+/VASA+co-localization on the basal layer of germ cell depleted seminiferous tubules in MOCK and i6F transplanted testis. **(B)** Percentage of tubules containing NuMA+/VASA+ cells. **(C)** Average number of NuMA+/VASA+ cells per tubule showing colonizing cells. **(D)** Efficiency of colonization per 10e5 injected cells in i6F transplanted testis (n = 5 testes). **(E)** Illustrative pictures showing NuMA/5mC co-localization and **(F)** NuMA/5hmC co-localization in transplanted testis. Data is presented as mean +/− SEM. Periphery of tubule cross-sections are highlighted by dashed lines. White arrows indicate colonizing human cells. Scale bar represents a distance of 50 μm.

**Figure 5:**
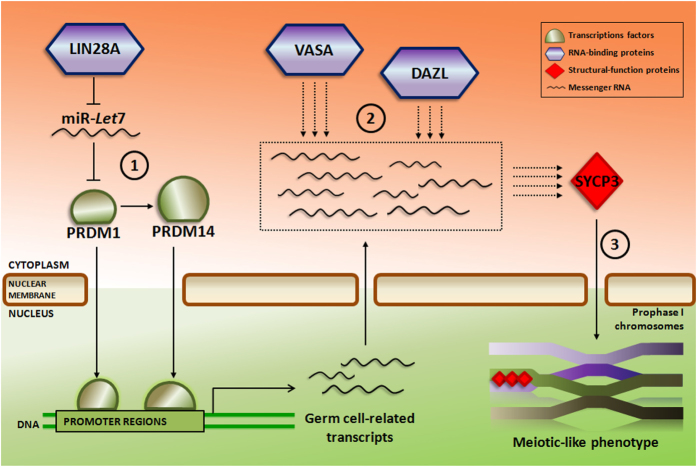
Proposed model for the germ line conversion and meiotic induction by i6F. The repressing effect of the RNA-binding protein LIN28A over the miRNA Let-7 allows that the transcription factors PRDM1 and PRDM14 bind to their specific genomic targets and switch on a germ line transcription program that generates a pool of mRNA transcripts (1). On the other hand, the RNA-binding proteins DAZL and VASA control the meiotic entry of the germ cell-like induced cells thanks to their regulatory functions over the pool of mRNA transcripts (2), at the same time that they (or probably other downstream translated proteins) organize the meiotic prophase I through the structural protein of the synaptonemal complex SYCP3 (3).

Immunostainings for co-localization of VASA and human NuMA^32^ were performed in frozen sections to detect human germ cells within the mouse testes as previously described^27^,^33^,^34^ (Fig. 4A and Supplementary Figure S6C). In 5 out of 8 transplanted testis, we detected double positive VASA+/NuMA+ cells. On average, 3.5% of tubule cross-sections showed colonizing donor cells located in the basal layer of both germ cell-depleted tubules and in tubules showing spontaneous recovery of the mouse spermatogenesis, with an efficiency of 0.76 colonizing cells per 10^5^ injected cells (Fig. 4B–D). Also, we observed that the engrafted cells stained for DAZL (Supplementary Figure S6D), UTF1 (Supplementary Figure S6E), 5mC (Fig. 4E) and 5hmC (Fig. 4F).

## Discussion

Several groups have reported the *in vitro* derivation of human germ cells from human embryonic stem cells (hESCs)^5^,^7^, induced pluripotent stem cells (iPSCs)^6^,^8^,^9^,^10^,^27^,^34^,^35^, and even from human skin-derived stem cells^11^. Also, based on the success of reprogramming terminally differentiated cells into a pluripotent state by defined factors^36^, several publications have reported the direct conversion of fibroblasts into other somatic lineages^37^,^38^,^39^,^40^,^41^. Based on this background, here we present a proof of concept that it is also possible to convert human somatic cells into a meiotic germ cell-like phenotype by inducing them with key gene regulators.

Trying to mimic the mesodermal origin of the germ line *in vivo*, we used two mesodermal human cultures of male foreskin fibroblasts and mesenchymal cells from bone marrow to conduct our experiments. Transduction of cells with a combination of 12 or 6 germ line-related factors resulted in a morphological change into cell clumps that was accompanied by the up-regulation of the epithelial marker CDH1 resembling a mesenchymal-to-epithelial transition (Supplementary Figure S3A,B), as previously described in SSCs derived from human testes^23^. Since clumps retained the expression of most of induced genes whereas the whole population of transduced cells showed a clear decrease of their expression along time, we hypothesize that clumps may be the germ cell-like phenotype resulting from i12F/i6F transduction (Supplementary Figure S1B). From a transcriptomic wide point of view, our results agree with a deep alteration of the cell cycle and chromatin structure in treated cells, as well as an activation of the TGFβ and LIF/STAT3 pathways (Supplementary Table S1).

In agreement with recent reports describing the transcriptomics of early human germ line development *in vivo*^42^ and *in vitro* from human pluripotent cells^43^, significant up-regulation of T (Brachyury), SOX17 and the naïve pluripotency marker TFCP2L1 was observed in i6F clumps, but also CDH1 and STELLA (Fig. 1C and Supplementary Figure S3B), indicating the presence of a PGC-like phenotype (Fig. 1C). However, although not significant in some cases, subsequent gene expression analysis showed an initial up-regulation of the spermatogonial marker GFRA1 that was gradually lost along the culture period, at the same time that meiotic (PIWIL2) and post-meiotic (TNP2, ACR and PRM1) markers raised along time in cell clumps (Fig. 1D and Supplementary Figures S3C and S5D). These observations, together with the localization of the spermatogonial markers PLZF, UTF1, VASA, DAZL and HIWI within the induced cell clumps (Fig. 1E), suggest the existence of a pre-meiotic germ cell-like population within the induced cultures that spontaneously differentiates into a meiotic-like phenotype along time. The co-existence of germ cell-like cells at different maturation stages within clumps is not totally unexpected since several factors expressed at different stages of germ line development *in vivo* were co-transduced in induced cells. Interestingly, we also found that fibroblastic cells surrounding i6F clumps expressed the Sertoli marker FSHR, and some cells within clumps expressed the stereidogenic Leydig marker 3βHSD and the Sertoli cell marker SOX9 (Supplementary Figure S7B), suggesting the possibility that some cells may acquire gonadal support properties that drive the maturation of germ cell-like cells within clumps.

In regard of the presence of pre-meiotic germ cell-like cells within i6F clumps, we aimed to establish pure germ cell-like lines from single cells coming from the clump sub-populations. However, picked cells did not progress *in vitro*. The reason for this is intriguing and we hypothesize that it may be due to the fact that cells within the clumps spontaneously enter into a meiotic cell cycle that results in the formation of haploid cells that cannot be further maintained *in vitro*. However, future studies should aim to address this point.

The optimized i6F cocktail resulting from the screening among the initial 12 factors included the two transcription factors PRDM1 and PRDM14 together with the RNA-binding proteins LIN28A, DAZL, VASA, and the structural protein of the synaptonemal complex SYCP3. PRDM1, PRDM14 and LIN28A are implicated in the core network that regulates the escape from the somatic fate during the germ line specification in the E6.5-E7.5 mice embryo *in vivo*^3^, but also *in vitro* by the overexpression of Blimp1 (Prdm1), Prdm14 and its downstream target Tfap2c on mouse epiblast-like cells (EpiLCs)^44^. DAZL and VASA have also been described as essential for the germ cell survival and meiotic progression *in vivo*^16^,^18^,^19^,^45^ and *in vitro*^7^,^8^,^9^,^27^,^34^. Indeed, a recent report already indicated that DAZL ectopic expression over goat MSCs enhanced their transdifferentiation into germ cells^46^. Based on these observations, we hypothesize that in our direct conversion model, the meiotic-like phenotype could be explained by the regulatory functions of the RNA-binding proteins DAZL and VASA on the pool of background transcripts induced by the induction with the transcription factors PRDM1 and PRDM14, also up-regulated by LIN28A (Fig. 5). However, it was surprising to learn that SYCP3 resulted essential for the meiosis initiation in induced cells. In accordance with previous data reporting the potential role of DAZL regulating the proper translation of VASA^47^ and SYCP3^48^ in mouse germ cells, we hypothesize that i6F factors are essential for the correct synaptonemal complex assembly since most of the cells subjected to SYCP3 overexpression alone or in combination with other factors apart of that included in the cocktail formed aberrant meiotic patterns (Supplementary Figure S4C).

According to our observations in meiotic and post-meiotic marker expression (Fig. 1), analysis of induced cells showed a subpopulation of cells that was already involved in a meiotic-like prophase I, based in their SYCP3 nuclear pattern. Meiotic-like nuclei were able to achieve proper chromosomal synapsis and even DSB phenomena as observed by the proper co-localization of SYCP3 with SYCP1 and γH2A.X. Although with very low efficiency, further ploidy analysis finally confirmed that some of meiotic nuclei were able to complete meiosis (Fig. 2). The low efficiency of meiotic entry of induced cells may be a direct consequence of the artificial nature of induced cells. However, since the transduction rates in our experiments, measured by GFP signal, ranged from 60 to 80%, it is possible that the high number of non-transduced cells within cultures may mask our results. Interestingly, we observed a higher incidence in the formation of female haploid cells (this is, haploid cells that kept the X chromosome) in all i12F/i6F conditions (Supplementary Tables S2 and S3). The reason for this trend to form female haploid cells is intriguing and ensures future research in a possible higher genomic stability compared to male haploid cells. Finally, we found a relative enrichment of haploid cells in clumps compared to the total population (Supplementary Figure S4F), suggesting that haploid cells are preferentially localized within clumps.

One of the key features of germ cells is the epigenetic reprogramming that they undergo during their development *in vivo*^13^. In contrast with the deep changes found in the transcriptomic analysis, methylation arrays indicated very slight differences between MOCK controls and induced samples (Fig. 3C), indicating that most of treated cells did not change their epigenetic profile at the methylation level. However, putative 1N sorted cells enriched for post-meiotic cells (Supplementary Figure S4D), showed a significant loss of methylation for paternally imprinted DMRs, but an increase in maternally imprinted ones (Fig. 3D). This result suggests that only post-meiotic cells may have acquired an epigenetic profile closer to the one expected from female oocytes. Since the chromosomal sex of the original primary cultures employed in this study was 46, XY, our results may correlate with previous reports suggesting that sexual determination of germ cells depends on the gonadal niche, being the female sex determination the predetermined destiny of germ cells^49^. This hypothesis may also be corroborated by the significant up-regulation of the follicle-specific markers GDF9 and ZP1 in i6F clumps (Supplementary Figure S7A,B). Finally, we detected a clear up-regulation in the expression of the TET-mediated de-methylation pathway members in i6F induced cells and especially in the case of clumps (Fig. 3B,C), suggesting that the TET-mediated de-methylation pathway is active in i6F induced cells as recently reported to occur in the human germ line *in vivo*^42^,^50^,^51^. However, it is possible that our results overestimate the percentage of methylation in induced cells since bisulphite conversion does not distinguish between 5mC and 5hmC. Despite this, and even that expression arrays also showed the up-regulation of genes involved in chromatin and histone modifications that suggest an epigenetic switch in induced cells (Supplementary Table S1), further efforts are necessary to shed light on the epigenetic status of the germ cell-like cells generated in our model.

Finally, we sought to test if i6F induced cells were able to colonize the spermatogenic niche in the same way that human SSCs do^30^,^31^. We found cells that a small fraction of i6F induced hFSKs were able to colonize the spermatogenic niche, indicating the existence of a small fraction of cells within i6F culture with a spermatogonial-like phenotype (Fig. 4A). However, the colonization efficiency of the induced germ cell-like cells generated in our model is low when compared with the efficiencies previously reported by others using similar techniques. Hence, we found an average of 3.5% of tubules containing NuMa+/VASA+ cells compared with a range of 20–30% of tubules in reports that transplanted human iPSCs^27^,^34^,^52^,^53^; In the same way, here we report a colonizing efficiency of 0.8 cells per 10e5 transplanted cells, compared with a wide range of efficiencies between 2.9 and 10 in reports where human testicular cell suspensions were transplanted^54^,^55^,^56^. Again, we can not discard that the low colonization efficiency observed in our model compared with other reports may be a direct consequence of the high number of non-transduced cells within transplanted cultures. Apart of VASA, engrafted cells expressed the spermatogonial marker UTF1 (Supplementary Figure S6D), and stained positive for 5hmC (Fig. 4F), indicating that TET-mediated de-methylation events continued in induced cells after transplantation. Future research will address if the xenotransplantation of i6F induced cells into the spermatogenic niche can help them to acquire the correct epigenetic status that they seem unable to acquire *in vitro*.

Germ cells are characterized by their ability to reduce their chromosomal charge during meiosis, and to erase epigenetic modifications accumulated during the lifespan to subsequently re-establish their sex-specific epigenetic profile. Our results obtained by inducing the exogenous expression of a combination of selected key germ cell developmental factors over human somatic cells demonstrate the ability of human male somatic cells to directly convert into a meiotic germ cell-like phenotype. Since some of the genes that we used in both i12F and i6F cocktails are expressed at different stages of mammalian germ line development, an inducible model for the activation of each of the transduced genes may increase the effectiveness in our model and future research should aim to test it. Nevertheless, our study must be considered an initial attempt to create a model for the direct conversion of somatic cells from patients with gamete production problems into germ cells *in vitro,* opening the field to discover new cell therapies to treat them in the future.

## Methods

### Cell culture

Conventional growth medium, DMEM/F12 supplemented with 10% FBS (Life Technologies) and 2mM L-glutamine (Millipore) was used to grow human foreskin fibroblasts (hFSK (46, XY), CRL-2429, ATTC) and human mesenchymal cells (hMSC (46, XY), PT-2501, Lonza). 24 hours after transduction, both MOCK and cells transduced with the different factors combinations were maintained in germ cell medium (GC-M), consistent on DMEM/F12 supplemented with 20% KnockOut Serum Replacement (all from Life Technologies), 2 mM L-glutamine, 0.1 mM nonessential amino acids, 0.1 mM 2-mercaptoethanol (Millipore), and supplemented with 1000 U/ml human recombinant Leukemia Inhibitor Factor (LIF, Sigma Aldrich), 20 ng/mL recombinant human Glial-Derived Neurotrophic Factor (GDNF, Peprotech), 5 μM Forskolin (Sigma Aldrich), 10 ng/ml basic Fibroblast Growth Factor (bFGF, Life Technologies), 5 ng/mL Stem Cell Factor (SCF, Peprotech) and 2 μM retinoic acid (Sigma Aldrich) at 37 °C, 5% CO_2_. All the experiments were performed at least in triplicate using low passages between passage 3 and 8 of initial primary cultures.

### Construction of expression vectors, lentiviral packaging and transduction

The Consensus Coding Sequences (CCDSs) of transgenes were isolated from reverse transcribed adult human testis total RNA (Clontech) and isolated PCR products were cloned into the lentiviral backbone pLenti7.3-V5 (Life Technologies) (Supplementary Figure S1C) using the Gateway system (Life Technologies). Lentiviral particles were generated by transient co-transfection of the lentiviral backbones containing the cloned CCDSs with the lentiviral packaging plasmids Delta8.9 and VSV-G (Addgene) using Lipofectamine 2000 (Life Technologies) over 293-T cells (CRL-11268, ATCC). Lentiviral titers from all 12 cloned CCDSs were 100000 TU/mL in average. The collected viral supernatants were supplemented with 8 μg/mL polybrene (Sigma Aldrich) and incubated with primary cultures. Remaining viral supernatants were washed off 24 h later and fresh GC-M was added the following day to both MOCK controls and cells transduced with the different combination of factors. GC-M was employed to keep transduced cells *in vitro* until their respective analysis.

### Reverse transcription and quantitative PCR assay

Total RNA was extracted according to manufacturer’s instructions via the RNeasy Mini Kit (Qiagen). Next, 500–1000ng of total RNA was converted into cDNA with oligo-dT by the Advantage RT-for-PCR kit (Clontech) following the manufacturer’s instructions. Transcription levels were determined by using a Lightcycler 480 (Roche) in triplicate reactions and normalized to the average of the housekeeping gene RPL19 with the 2^−ΔΔCt^ method. Primers used to detect gene expression are described in Supplementary Table S4. The average fold change relative to MOCK controls of four samples of human testis tissue from post-vasectomized patients was employed as a positive control for germ line markers. Informed consent was obtained from all subjects and all experiments involving human tissue were performed in accordance with approved guidelines and regulations upon the corresponding approval of the ethical committee of the Instituto Valenciano de Infertilidad (IVI) (Project Reference: 1203-C-100-JM).

### Expression arrays

RNA samples from 14 days post-transduction cultures were analyzed with the Whole Human Genome Oligo Microarray (Agilent GE 4 × 44K Human v2 microarrays, Agilent Technologies). Total RNA was processed according to manufacturer’s microarray instructions. Gene expression scanned values were preprocessed, normalized, and statistically analyzed. The half-background median intensity values were subtracted from the average intensity of each spot, and were normalized between arrays using the quantile method implemented in the Bioconductor (www.bioconductor.org) LIMMA package run under the R software (www.r-project.org). Probe sets belonging to the same gene were merged by median and transformed to the logarithmic scale (log2) giving rise to a final matrix of 25,512 genes by sample. The microarray analysis was performed using Babelomics software version 4.3 (babelomics.bioinfo.cipf.es)^57^. Exploratory analysis of whole gene expression data was performed to check the behavior of samples using Principal Component Analysis and UPGMA clustering using Euclidean distance among samples. Functional enrichment for differential expressed genes was performed with FatiGO^58^ a widely used SEA implementation, which is included in the Babelomics^57^ web-based package using the Gene Ontology (GO)^59^ and Reactome curated databases^60^. The significant over-represented terms related to human genome comparison (adjusted p-value < 0.05) were considered. Results were validated by qPCR for three of the top up-regulated and down-regulated genes (data not shown). All microarray data have been deposited at the NCBI Gene Expression Omnibus (GEO) under accession number GSE64479.

### Immunocytochemistry

For frozen sections, before immunostainning tissue was placed in 30% Sucrose at 4 °C overnight, frozen in Optimum Cutting Temperature (OCT) compound (VWR) and sections were prepared at 8 μm thickness. Cells and/or tissue sections were fixed with 4% paraformaldehyde for 15 minutes at room temperature. For 5mC and 5hmC staining, denaturation of DNA was carried out by treating samples with 4M HCl/0.1% Triton X-100 for 10 min and immediately neutralized with 100mM Tris/HCl (pH 8.5) for 30 minutes. In the case of intra-cellular antigens, cells were permeabilized with 1% Triton X-100 (Sigma Aldrich). Samples were blocked with 4% of serum of the animals were the secondary antibodies were raised for one hour at room temperature, followed by incubation of primary antibodies in 1% of blocking serum overnight at 4 °C. A list with the primary antibodies employed is showed in Supplementary Table S5. A dilution of 1:1,000 of secondary Alexa fluor antibodies were incubated for one hour in darkness at room temperature prior to mount the slides with ProLong Gold antifade reagent with DAPI (Life Technologies). Negative controls were performed by the omission of the first antibody. Immunostainings over human testis frozen sections with the same antibodies were employed as positive controls. Informed consent was obtained from all subjects and all experiments involving human tissue were performed in accordance with approved guidelines and regulations. Mounted slides were visualized using a fluorescence microscope.

### Meiotic spreads

Meiotic spreads were performed as previously described with modifications^7^,^9^. Briefly, cells were collected and resuspended in hypoextraction buffer (30 mM Tris (Sigma Aldrich), 50 mM Sucrose (Sigma Aldrich), 17 mM Citric acid (Sigma Aldrich), 5 mM EDTA (Life Technologies), 0.5 mM DTT (Sigma Aldrich) and 0.5 mM PMSF, a protease inhibitor (Thermo Scientific); pH8.2) for 30 min and then put onto slides. The slides were fixed with 1% paraformaldehyde overnight and permeabilized 5 min with 0.04% photoflo (KODAK), followed by blocking 60 min with 4% donkey serum in 1% BSA, 0.1% Triton-X100 in PBS. Then, primary antibodies (Supplementary Table S5) were incubated at 37 °C in a humid chamber. Slides were washed with 1% BSA + 0.1% Triton-X100 in PBS prior to secondary antibodies incubation at 37 °C. ProLong Gold antifade reagent (Life Technologies) was applied and 1,000 cells/condition were counted under fluorescence microscope in triplicate experiments.

### Cell sorting and fluorescence *in situ* hybridization (FISH)

Single cells were fixed with 70% ice cold ethanol overnight and then resuspended in a solution containing 0.2 mg/ml RNase A and 0.02 mg/ml propidium iodide (Life Technologies) in BD Perm/Wash buffer (BD). Putative haploid cells were sorted using a MoFlo (Modular Flow Cytometer; Beckman Coulter, Dako) jet-in-air high speed sorter. Sorted cells were collected and fixed with Carnoy’s fixative (1:3 acetic acid:methanol) and air dried. The slides were then dehydrated in 70%, 80% and 100% ethanol 1 min each. FISH probes against chromosomes 18 (D18Z1,Kreatech, KI-20018-B), X and Y ((SE X(DXZ1)/ SEY (DYZ3), Kreatech, KBI-20030) were denatured on slides at 73 °C 5min and hybridized at 37 °C overnight. Additionally, centromeric probes for chromosomes 10 (aqua), 12 (green) and 3 (red) were employed to verify the ploidy of previously analyzed nuclei (Vysis). Slides were then washed with 2X SSC/NP-40, incubated with 0.4X SSC for 90 seconds at 73 °C, and then washed again in 2X SSC/NP-40. Prolong Gold antifade with DAPI was applied and between 200–1,000 cells/condition were counted under fluorescence microscope in triplicate experiments. Human sperm was employed as positive control. Informed consent was obtained from all subjects and all experiments involving human tissue were performed in accordance with approved guidelines and regulations.

### Molecular ploidy assessment by PCR and aCGH

Single cells were individually isolated and subjected to whole genome amplification (WGA) by the Sureplex DNA amplification system (BlueGnome) according to manufacturer’s instructions. Amplification quality was ensured by gel electrophoresis (Lonza, Rockland, USA). Two microlitres from each WGA mix were then subjected to the AMELOGENIN PCR employing a forward primer labeled with VIC in the presence of 5 mM MgCl_2_, 0.2 mM dNTPs, 10 pmole each primer, and 1 unit Platinum Taq polymerase (Life Technologies). PCR amplifications were initiated at 95 °C for 5 min followed by 45 cycles of 95 °C, 25 sec; 55 °C, 35 sec; and 72 °C, 35 sec. PCR products were finally analyzed in a 3130 Genetic Analyzer (Applied Biosystems). For aCGH analysis, WGA DNAs and control DNAs were labelled with Cy3 and Cy5 fluorophores following the manufacturer’s instructions. Labelling mixes were combined and hybridized on 24sure arrays (V2 and V3, BlueGnome, Cambridge, UK) for 6–12 hours. Fluorescence intensity was detected using a laser scanner (Powerscanner, TECAN, Mannedorf, Switzerland), and BlueFuse Multi software was used for data processing (BlueGnome, Cambridge, UK).

### Methylation arrays

We generated methylation datasets using the Illumina Infinium HumanMethylation450 BeadChip arrays, which simultaneously quantifies ~2% of all CpG dinucleotides. Bisulphite conversion of 600 ng of DNA was performed according to the manufacturer’s recommendations for the Illumina Infinium Assay (EZ DNA methylation kit, ZYMO, Orange, CA). The bisulphite-converted DNA was used for hybridization following the Illumina Infinium HD methylation protocol. We applied signal background subtraction and inter-plate variation was normalized using default control probes in BeadStudio (version 2011.1_Infinium HD). We discarded probes with a detection p-value >0.01 (n = 365). We also excluded probes that lack signal values in one or more of the DNA samples (n = 91). Furthermore we removed 26,819 probes since they are known to be influenced by SNP genotypes. Therefore 458,237 probes were used during the screening processes. For the analysis of known imprinted domains, probes mapping to the DMRs identified by Court and colleagues were directly analyzed^61^. To identify regions that changed with transfection of the transduced gene cocktail, an in-house bioinformatics pipeline (using R-package) was utilized to identify probes with **>**0.2 β (a change of 20% absolute methylation) between MOCK and experiment, with the additional criteria that prioritized loci if they had more than two probes within 1 kb. The circular heatmaps used to display the DNA methylation profiles were generated using Circus software.

### Bisulfite Sequencing

Genomic DNA was extracted via the QIAamp DNA Mini kit (Qiagen) and 100 ng/sample were processed using the Epitect Bisulfite Kit (Qiagen) according to manufacturer’s instructions. One microlitre of bisulphite-treated genomic DNA was PCR amplified following previously reported protocols^62^,^63^,^64^. The resultant PCR products were gel-extracted using the Qiaquick gel extraction kit (Qiagen) and cloned into TOPO TA vectors (Life Technologies). At least 20 clones were sequenced using ABI BigDye v3.1 dye terminator sequencing chemistry (Applied Biosystems) and ABI PRISM 3730xl capillary DNA analyzer for sequence analysis. CpG methylation analysis was performed by BiQ Analyzer software (Max Planck Institut Informatik).

### Germ cell xenotransplantion

All animal experiments in this study were previously approved by the Ethical Committee for Animal Wellness by the University of Valencia (REF: A1332863413145) and all experiments were performed in accordance with approved guidelines and regulations. Five to six-week-old immune NMRI deficient Nude males (RjOrl:NMRI-*Foxn1*^*nu*^*/Foxn1*^*nu*^) (JanvierLabs) were treated with 35 mg/kg of busulfan (Sigma Aldrich) by intra-peritoneal injection. Four weeks after busulfan treatment, 14 days-transduced cells were harvested and between 1 and 3 million of cells/testis were transplanted into the seminiferous tubules of recipient mice (n = 4 mice and 8 testes per experimental condition) via *vas deferens* injection as previously described^30^,^65^. Two months after transplantation, mice were sacrificed and their testes were processed for frozen sections as described above. We counted NuMA+/VASA+ double positive cells located on the basal layer of tubules of 15 serial sections per transplanted testis with a distance of 80 μm among them. The average number of double positive cells found by two independent researchers was divided by the number of injected cells in each testis and multiplied by 10^5^ to obtain the efficiency of colonization per 10^5^ injected cells.

### Statistics

Statistical analysis of RT-qPCR results and meiotic and FISH counts was performed with one-way ANOVA and t-student pair wise comparisons by SPSS software (SPSS Inc.). Significance was accepted at p < 0.05. For expression arrays, we applied the LIMMA test for deep screening analysis between groups (two-class comparisons), and compared the expression patterns from treated samples with i12F/i6F versus MOCK controls, obtaining adjusted *p*-values and fold changes for each comparison (FC higher than 2; adjusted *p*-value of less than 0.05); we corrected the *p* values using the false discovery rate accounting for multiple testing effects. Gene expression data fold change from significant genes was used to compare with RT-qPCR values (data not shown).

## Additional Information

**How to cite this article**: Medrano, J. V. *et al*. Human somatic cells subjected to genetic induction with six germ line-related factors display meiotic germ cell-like features. *Sci. Rep.* **6**, 24956; doi: 10.1038/srep24956 (2016).

## Supplementary Material

Supplementary Information
