# Supplementary material for: Human somatic cells subjected to genetic induction with six germ line-related factors display meiotic germ cell-like features
Source: Sci Rep. 2016 Apr 26;6:24956. doi: 10.1038/srep24956 (PMC4844986; doi:10.1038/srep24956)
Supplement: Supplementary Information [file srep24956-s1.pdf]

Human somatic cells subjected to genetic induction with six germ line-related factors display meiotic germ cell-like features

Jose V. Medrano[1,2,+], Ana M. Martínez-Arroyo[1,+], Jose M. Míguez[1], Inmaculada Moreno[3], Sebastián Martínez[1], Alicia Quiñonero[1], Patricia Díaz-Gimeno[1], Ana I. Marqués-Marí[4], Antonio Pellicer[2], Jose Remohí[1] & Carlos Simón[1,3,5*]

[1]Fundación Instituto Valenciano de Infertilidad (FIVI), INCLIVA; Department of Pediatrics, Obstetrics and Gynecology, Valencia University; Valencia, 46015; Spain

[2]Fundación Instituto de Investigación Sanitaria La Fe; Valencia, 46015; Spain

[3]Igenomix S.L.; Paterna, 46980; Spain

[4]Valencia Node of the Spanish Stem Cell Bank, Prince Felipe Research Centre (CIPF) Valencia, 46012; Spain

[5]Department of Obstetrics and Gynecology, Stanford University; Stanford, CA 94305; USA

[+]These authors contributed equally to this work.

***Correspondence and reprint requests should be addressed to:** Prof. Carlos Simon at Fundación Instituto Valenciano de Infertilidad (FIVI), PARC CIENTIFIC UNIVERSITAT DE VALENCIA, Catedrático Agustín Escardino 9, 46980 Paterna (Valencia), Spain; Phone: +34 963903305; Fax: +34 963902522; E-mail: carlos.simon@ivi.es

1. Inventory of Supplementary Information

Supplementary Figure S1. Analysis of the expression of induced factors over hFSK cells.

Supplementary Figure S2. Screening among i12F factors over hFSK cells.

Supplementary Figure S3. Supplementary information regarding culture set up, transcriptomic characterization of induced hFSK cells.

Supplementary Figure S4. Supplementary data regarding meiotic progression analysis in hFSK induced cells.

Supplementary Figure S5. Supplementary data regarding culture phenotype, meiotic progression analysis and transcriptomics in hMSC induced cells.

Supplementary Figure S6: Supplemental data relative to xenotransplant experiments.

Supplementary Figure S7. Male and female gonadal specific marker analysis and antibody staining controls.

Supplementary Table S1. List of the top represented terms of the functional enrichment analysis over the i12F and i6F cultures compared to MOCK.

Supplementary Table S2. Summary of FISH-based ploidy counts in hFSK cells.

Supplementary Table S3. Summary of FISH-based ploidy counts in hMSC cells.

Supplementary Table S4. Primer sequences used to gene expression analysis by RT-qPCR.

Supplementary Table S5. List of the antibodies employed.

2. Supplementary Figures and Legends.

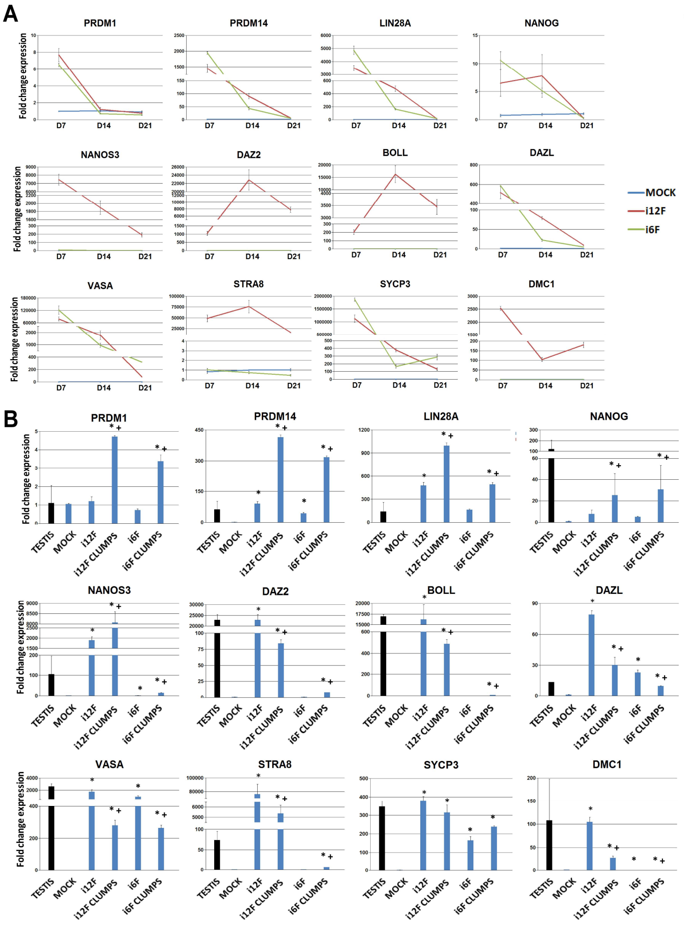

C Cloned Consensus Coding DNA Sequence (CCDS) from:

GENE	GenBank accession no.	Cloned amplicon length (bp)
PRDM1	NM_182907.2	2080
PRDM14	NM_024504.3	1720
LIN28A	NM_024674.4	634
NANOS3	NM_001098622.2	583
NANOG	NM_024865.2	922
DAZ2	NM_020363.3	1692
DAZL	NM_001351.3	892
BOLL	NM_197970.2	892
VASA	NM_024415.2	2179
STRA8	NM_182489.1	997
SYCP3	NM_001177949.1	715
DMC1	NM_007068.2	1027

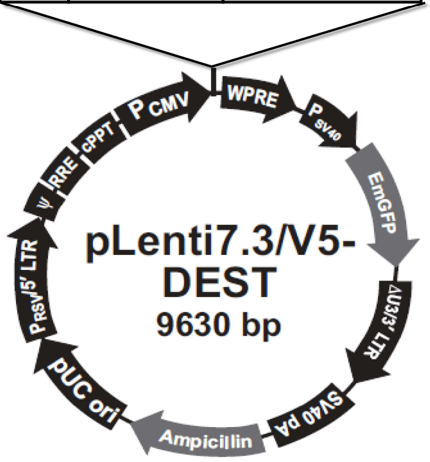

Supplementary Figure S1. Analysis of the expression of induced factors over hFSK cells. (A) Time course analysis of the mRNA expression of the transduced factors at 7, 14 and 21 days post-transduction in MOCK, i12F and i6F conditions (n=3). **(B)** RTqPCR expression analysis of the twelve overexpressed factors at day 14 post-transduction over i12F, i12 clumps, i6F and i6F clumps (n=3). Human testis cDNA physiological expression fold change relative to MOCK samples is also shown as a control. Data is presented as normalized fold change mean +/- SEM. (*) represent significant differences ($p<0.05$) with MOCK controls; (+) represents significant differences ($p<0.05$) between i12F/i6F conditions and their respective induced germ cell-like cell clumps. **(C)** Overview of the cloning methodology employed for the construction of lentiviral vectors. The table shows information regarding GeneBank

accession numbers, primers employed for the isolation of the cloned CCDSs and length of the amplicons. The map of the pLenti7.3/V5-DEST backbone with the site of insertion of cloned CCDSs is also shown.

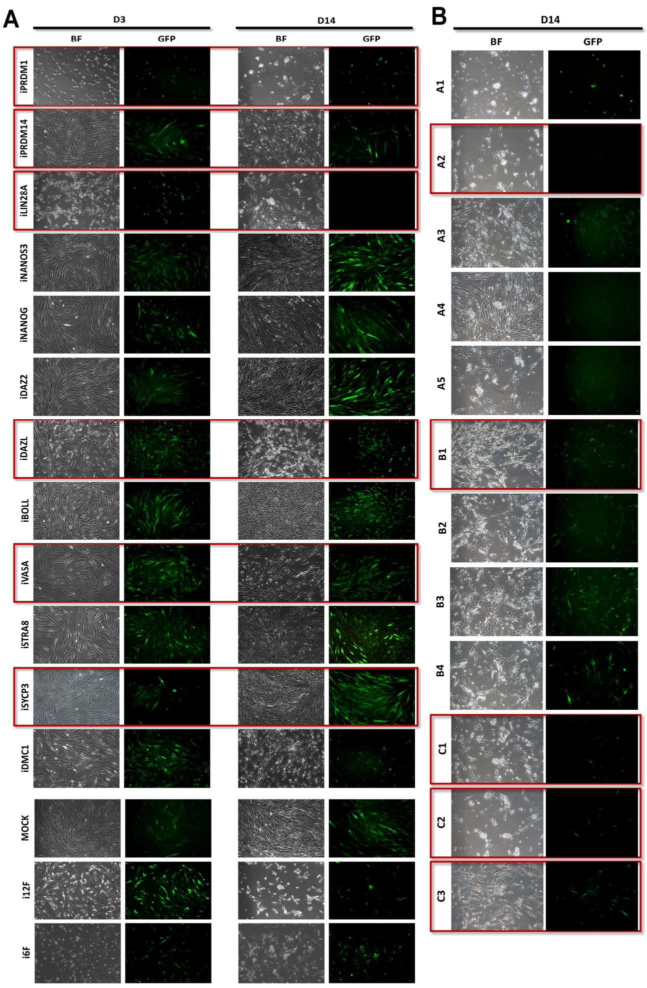

C

INDUCTORS	A1	PRDM1	PRDM14				
	A2	PRDM1	PRDM14	LIN28			
	A3	PRDM1	PRDM14	LIN28	NANOS3		
	A4	PRDM1	PRDM14	LIN28	NANOG		
	A5	PRDM1	PRDM14	LIN28	NANOS3	NANOG	
MADURATORS	B1	DAZL	VASA				
	B2	DAZL	VASA	STRA8			
	B3	DAZL	VASA	BOULE			
	B4	DAZL	VASA	STRA8	BOULE		
COMBINATIONS A+B	C1	PRDM1	PRDM14	LIN28	DAZL		
	C2	PRDM1	PRDM14	LIN28	DAZL	VASA	
	C3	PRDM1	PRDM14	LIN28	DAZL	VASA	STRA8

Supplementary Figure S2. Screening among i12F factors over hFSK cells. (A) Illustrative bright field and GFP fluorescence pictures at days 3 and 14 post-transduction of human fibroblasts subjected to the overexpression of each germ line-related factor alone and subjected to MOCK, i12F and i6F transduction. Scale bar represents a distance of 50µm. **(B)** Illustrative bright field and GFP fluorescence pictures of hFSK cells subjected to the overexpression of the most interesting combinations of factors among i12F that generated the formation of clumps. Red rectangles highlight the most efficient factors/combinations to induce the formation of clumps.

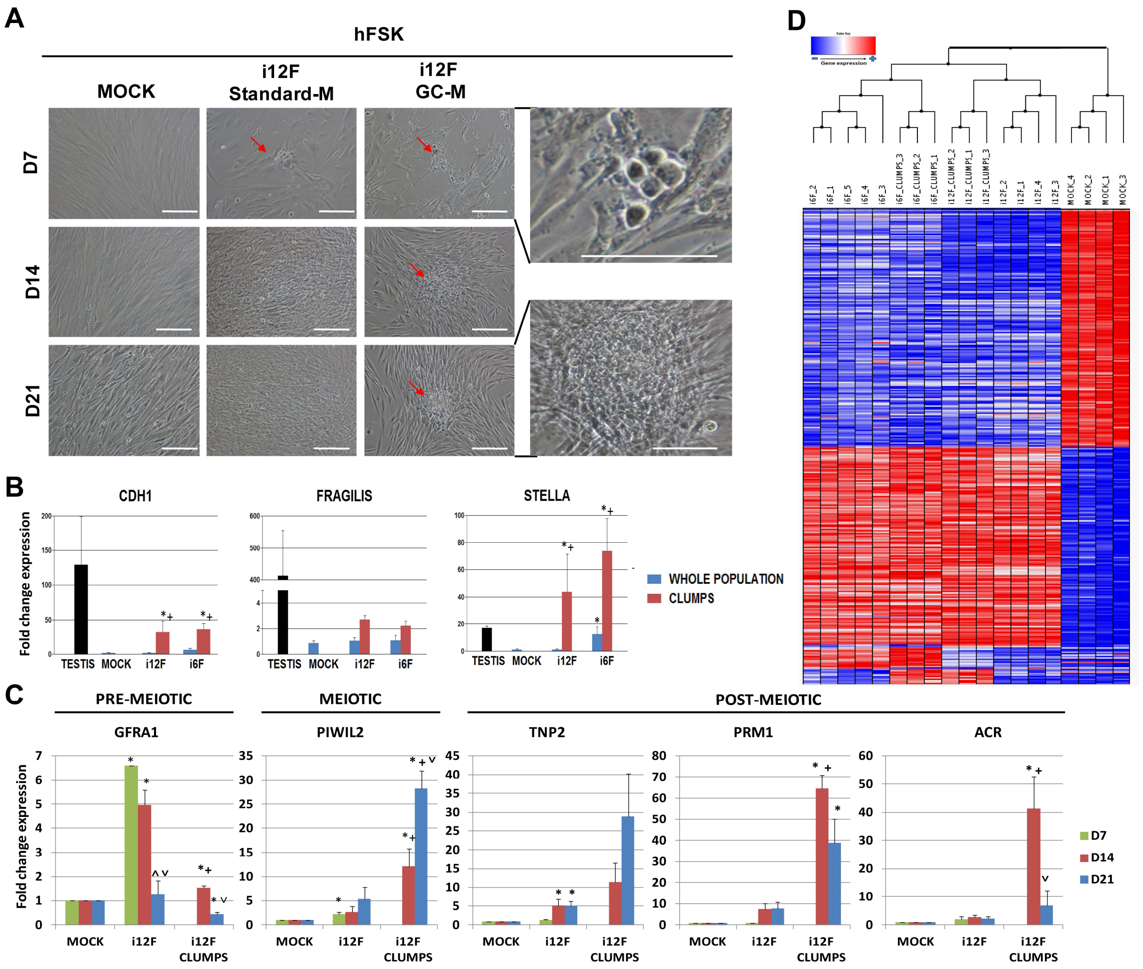

Supplementary Figure S3. Supplementary information regarding culture set up, transcriptomic characterization of induced hFSK cells. (A) Illustrative pictures at 7, 14 and 21 days post-transduction of the formation of induced germ cell-like cell clumps (indicated by red arrows) showing the morphology of primary cultures after transduction with the germ cell factors in standard and germ cell medium (GC-M). Higher magnifications correspond to hFSK cells at early (7 days) and late (21 days) time points post-transduction, respectively. **(B)** Expression analysis of the epithelial marker CDH1 and the early germ cell markers FRAGILIS and STELLA 14 days post-transduction with i12F/i6F transduced hFSK cells (n=3). **(C)** RT-qPCR expression analysis of the germ line markers *GFRA1*, *PIWIL2*, *TNP2*, *PRM1*, and *ACR* over i12F induced hFSKs at 7, 14 and 21 days post-transduction (n=8). **(D)** Unsupervised hierarchical clustering and heat map of differentially expressed genes in induced hFSKs.

Data is presented as normalized fold change mean +/- SEM. (*) represent significant differences ($p<0.05$) with MOCK controls; (+) represents significant differences ($p<0.05$) between i12F/i6F conditions and their respective induced germ cell-like cell clumps; (^) represent significant differences ($p<0.05$) with day 7 expression within sample groups; (v) represent significant differences ($p<0.05$) with day 14 expression within sample groups. Scale bar represents a distance of 50μm.

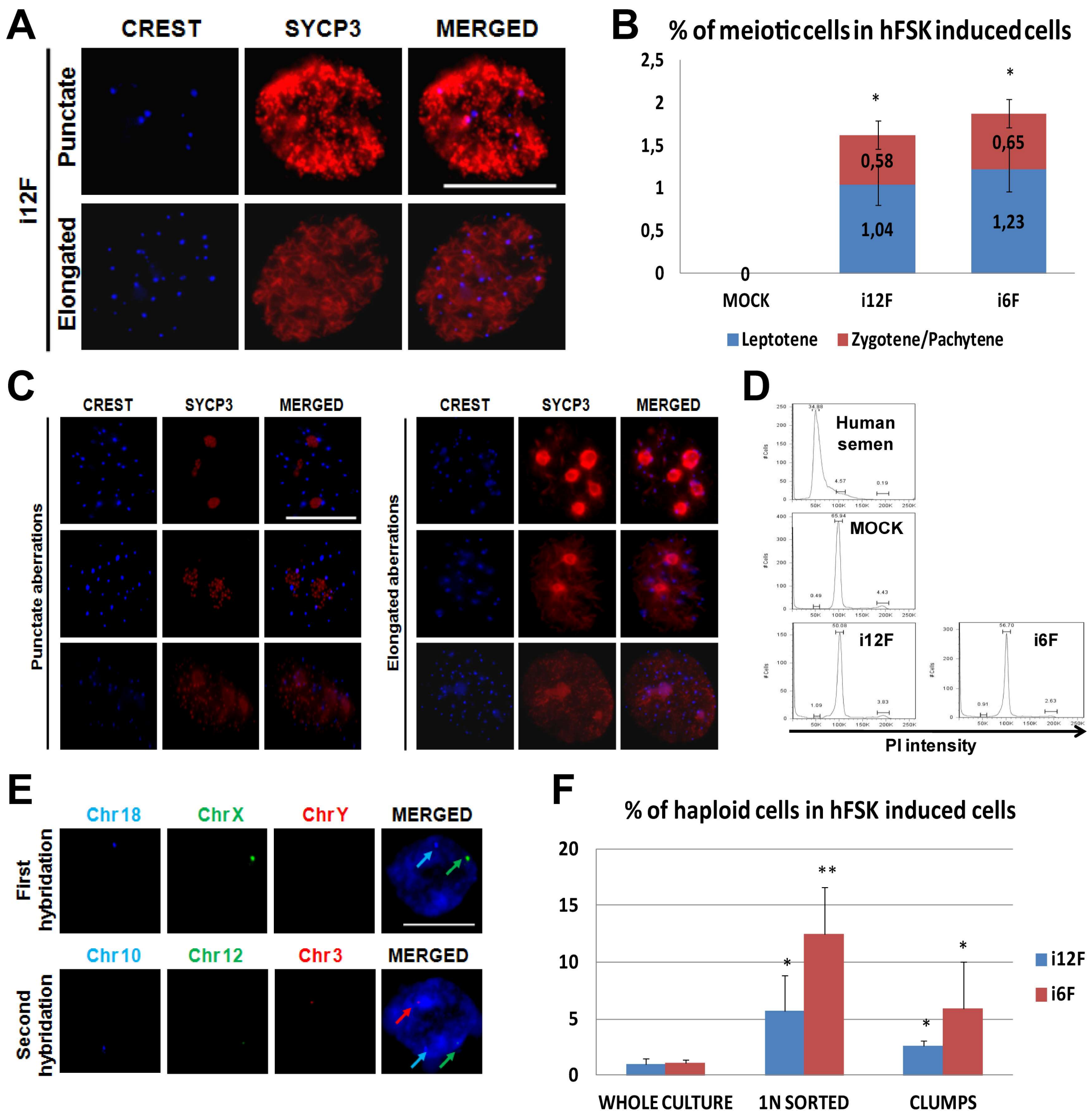

Supplementary Figure S4. Supplementary data regarding meiotic progression analysis in hFSK induced cells. (A) Illustrative pictures of the SYCP3 staining pattern over i12F transduced cells. **(B)** Percentage of punctate/elongated staining pattern found in i12F and i6F transduced hFSK cells (n=8). **(C)** Aberrant SYCP3 staining patterns found over transduced cells. **(D)** Representative DNA-fluorescence-activated cell sorting plots for the isolation of the putative 1N populations. Sperm control was used to set the sorting parameters. **(E)** Re-hybridation of chromosomes 18, X and Y FISH analyzed nuclei with centromeric probes for chromosomes 10 (aqua), 12 (green) and 3 (red) confirms haploidy of induced cells. **(F)** Percentage of haploid cells found in

i12F/i6F treated cells based on the FISH analysis with probes for the chromosomes 18, X and Y in hFSK cells (n=8). Data is presented as mean +/- SEM. (*) and (**) represent significant differences ($p<0.05$ and $p<0.01$, respectively) with MOCK and MOCK 1N sorted controls, respectively. Scale bars represent a distance of 10μm.

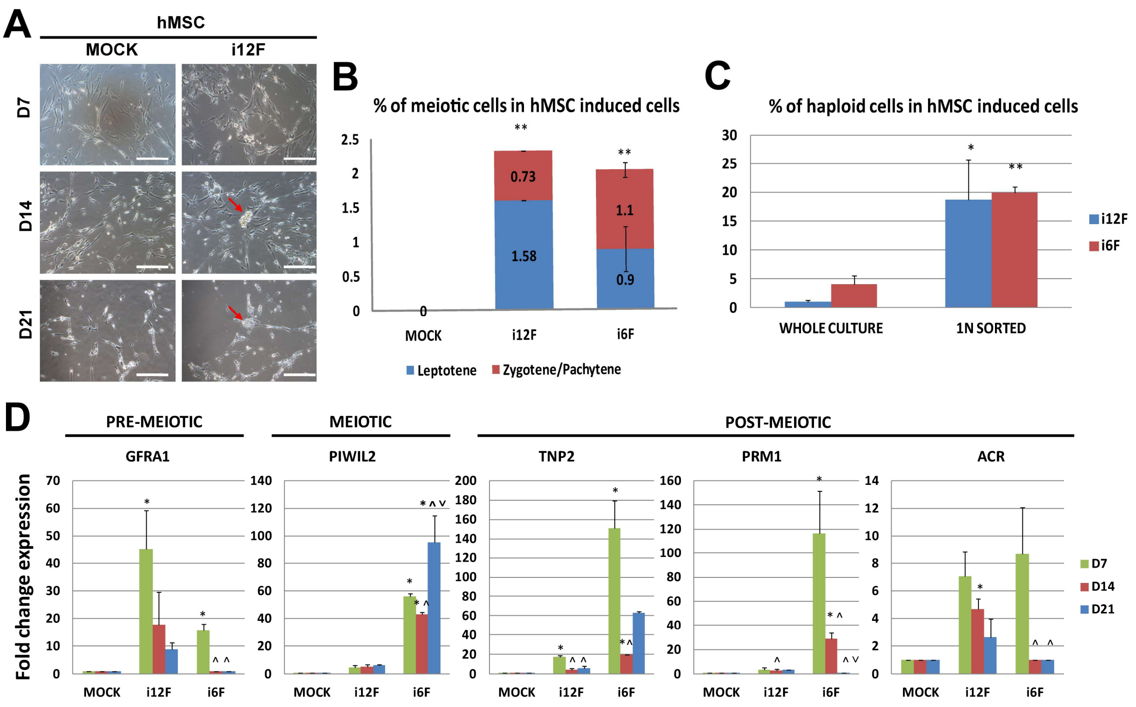

Supplementary Figure S5. Supplementary data regarding culture phenotype, meiotic progression analysis and transcriptomics in hMSC induced cells. (A) Illustrative pictures at 7, 14 and 21 days post-transduction of the formation of induced germ cell-like cell clumps (indicated by red arrows) showing the morphology of primary cultures after transduction with the germ cell factors in standard and germ cell medium (GC-M). Scale bars represent a distance of 10µm. **(B)** Percentage of punctate/elongated staining pattern found in i12F and i6F transduced hMSCs (n=4). **(C)** Percentage of haploid cells found in i12F/i6F treated cells based on the FISH analysis with probes for the chromosomes 18, X and Y in hMSC cells (n=4). Data is presented as mean +/- SEM. (*) and (**) represent significant differences (p<0.05 and p<0.01, respectively) with MOCK and MOCK 1N sorted controls. **(D)** RT-qPCR expression analysis of the germ line markers *GFRA1*, *PIWIL2*, *TNP2*, *PRM1*, and *ACR* over i12F/i6F induced hMSCs at 7, 14 and 21 days post-transduction (n=8). Data is presented as normalized fold change mean +/- SEM. (*) represent significant differences (p<0.05) with MOCK controls; (+) represents significant differences (p<0.05) between i12F/i6F

conditions and their respective induced germ cell-like cell clumps; (∧) represent significant differences ($p<0.05$) with day 7 expression within sample groups; (∨) represent significant differences ($p<0.05$) with day 14 expression within sample groups.

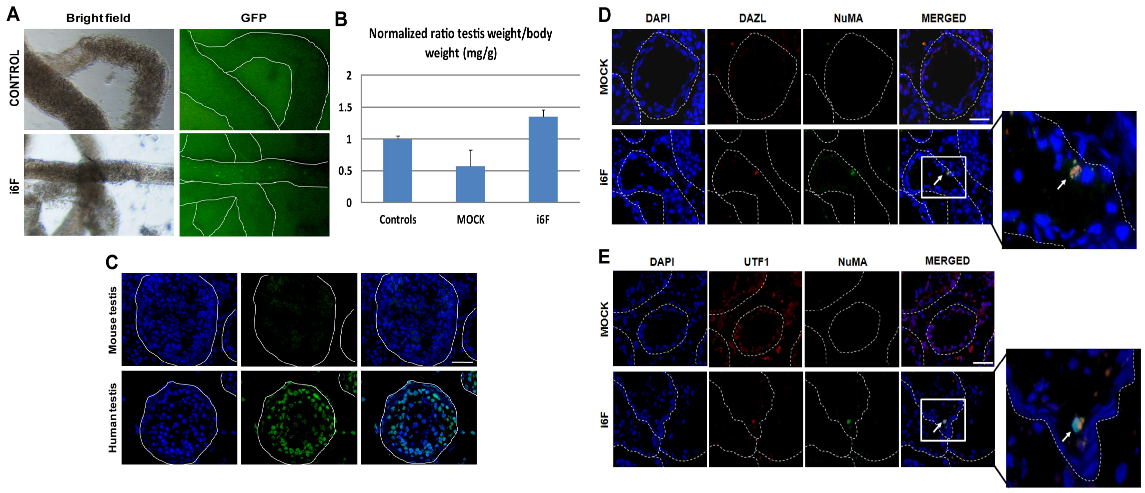

Supplementary Figure S6. Supplementary data relative to xenotransplant experiments. (A) Illustrative pictures showing the presence of small GFP dots corresponding to putative colonizing cells within seminiferous tubules transplanted with i6F cells. **(B)** Normalized ratio of testis weight in milligrams with the body weight in grams of transplanted mice. Controls were busulfan treated but not transplanted mice (n=8 testes). **(C)** Illustrative staining of NuMA antibody localizing in the nucleus of human testicular cells but not in mouse. **(D)** Illustrative pictures showing co-localization of NuMA and DAZL and **(E)** NuMA/UTF1 in the nuclei of engrafted cells. Data is presented as mean +/- SEM. (*) represent significant differences ($p<0.05$) with non transplanted controls; (+) represents significant differences ($p<0.05$) with MOCK transplanted testes. Periphery of tubule cross-sections are highlighted by dashed lines. White arrows indicate colonizing human cells. Scale bar represents a distance of 50μm.

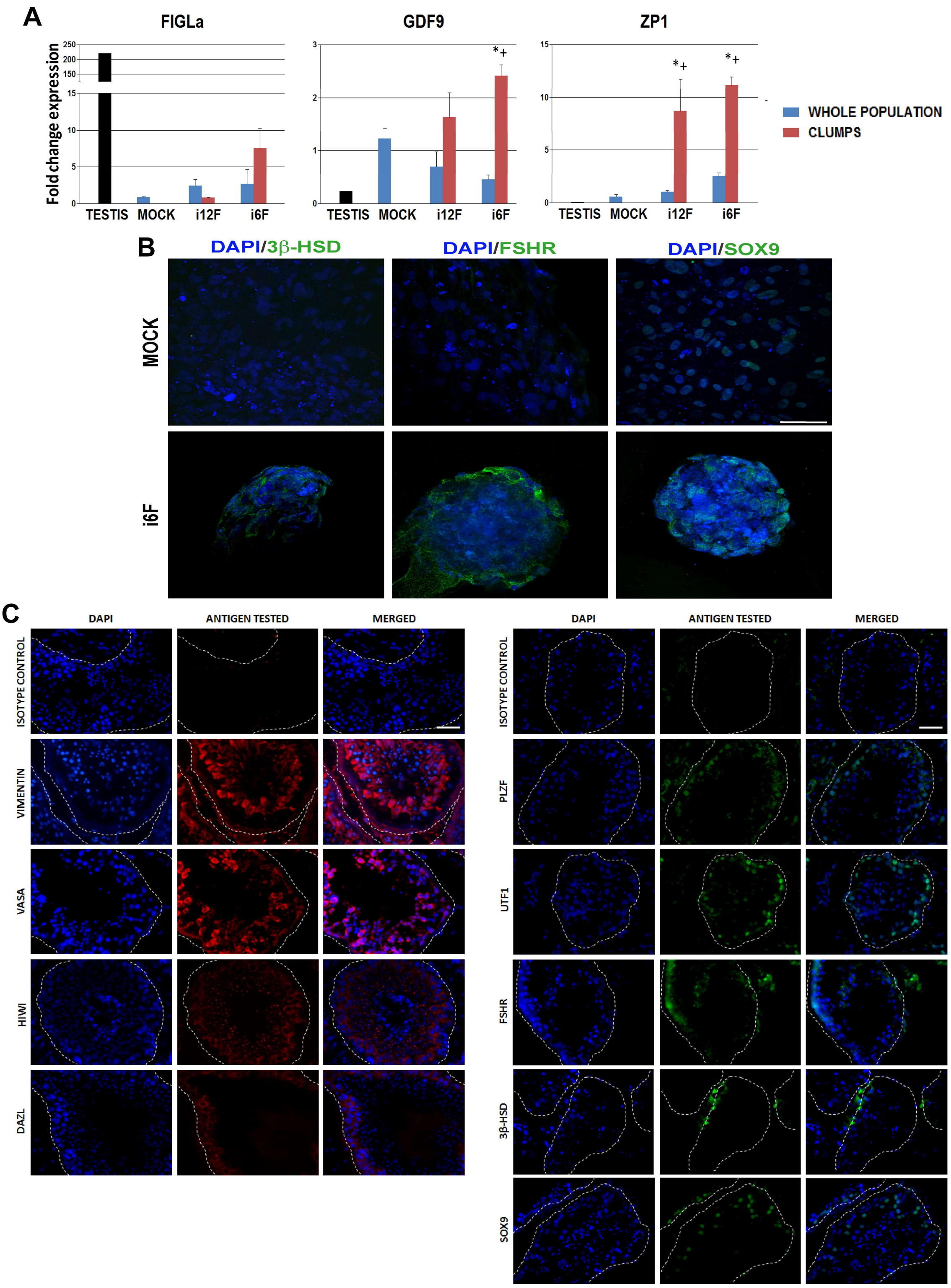

Supplementary Figure S7. Male and female gonadal specific marker analysis and antibody staining controls. (A) RTqPCR analysis of the expression of the follicle-specific developmental markers FIGLα, GDF9 and ZP1 over 12F/i6F induced hFSKs 14 days post-transduction. **(B)** Illustrative pictures of immunofluorescencent stainings for

the Leydig cell marker 3βHSD and the Sertoli cell markers FSHR and SOX9 over MOCK and i6F clumps from hFSK cells. **(C)** Antibody staining controls for primary antibodies over testicular sections. Data is presented as normalized fold change mean +/- SEM. (*) represent significant differences ($p<0.05$) with controls; (+) represents significant differences ($p<0.05$) between i12F/i6F conditions and their respective induced germ-cell like cell clumps. Scale bar represents a distance of 50µm.

3. Supplementary Tables.

Supplementary Table S1. List of the top represented terms of the functional enrichment analysis over the i12F and i6F cultures compared to MOCK. Term annotation % represents the ratio of represented genes in the list of the differentially expressed genes; GO, Gene Ontology; BP, Biological Process; MF, Molecular Function; CC, Cellular Compartment.

Treatment	Term	GO level	Term annotation %	Annotated ids	Adjusted p-value
i12F/i6F	retinoic acid metabolic process (GO:0042573)	BP	1.3	CYP26B1,CYP26A1,RBP1,MAFB	1.38352E-3
i12F/i6F	negative regulation of cell growth (GO:0030308)	BP	2.28	CGREF1,ENPP1,CDKN2C,BCL2,APBB2,INHBA,GREM1	4.55732E-3
i12F/i6F	mesenchymal cell development (GO:0014031)	BP	1.95	HGF,BCL2,GDNF,CYP26A1,EDN1,MAFB	4.88577E-3
i12F/i6F	negative regulation of transcription factor activity (GO:0043433)	BP	1.63	ID1,ID3,ID2,HMOX1,MAFB	5.68985E-3
i12F/i6F	transcription initiation (GO:0006352)	BP	2.61	HIST1H4D,HIST1H4L,HIST1H4F,HIST1H4C,HIST1H4J,HIST1H4E,HIST1H4I,MAFB	6.80627E-3
i12F/i6F	regulation of protein kinase activity (GO:0045859)	BP	4.23	GAP43,ADORA2B,PKMYT1,RGS4,HGF,CDKN2C,CDKN1C,ERN1,WNT7B,ITGA1,FPR1,EDN1,MAFB	1.60413E-2
i12F/i6F	positive regulation of DNA replication (GO:0045740)	BP	1.3	AREG,IL6,CSF2,MAFB	1.71113E-2
i12F/i6F	positive regulation of MAP kinase activity (GO:0043406)	BP	2.28	ADORA2B,HGF,WNT7B,ITGA1,FPR1,EDN1,MAFB	1.9534E-2
i12F/i6F	transforming growth factor beta receptor signaling pathway (GO:0007179)	BP	1.95	ID1,GDF10,FMOD,CDKN1C,CHST11,MAFB	2.29673E-2
i12F/i6F	regulation of MAP kinase activity (GO:0043405)	BP	2.61	ADORA2B,RGS4,HGF,WNT7B,ITGA1,FPR1,EDN1,MAFB	2.30361E-2
i12F/i6F	positive regulation of hormone secretion (GO:0046887)	BP	1.3	INHBA,CRH,EDN1,MAFB	2.56881E-2
i12F/i6F	anti-apoptosis (GO:0006916)	BP	3.26	CLU,HGF,CDC2,BCL2,GDNF,FN1,HMOX1,IL6,CSF2,MAFB	3.23274E-2
i12F/i6F	ovarian follicle development (GO:0001541)	BP	1.3	BCL2,LFNG,INHBA,MAFB	3.41505E-2
i12F/i6F	positive regulation of protein kinase activity (GO:0045860)	BP	2.93	GAP43,ADORA2B,HGF,ERN1,WNT7B,ITGA1,FPR1,EDN1,MAFB	3.80244E-2
i12F/i6F	negative regulation of phosphorylation (GO:0042326)	BP	1.3	ENPP1,CDKN2C,CDKN1C,INHBA	4.06159E-2
i12F/i6F	regulation of cell migration (GO:0030334)	BP	3.75	SST, F10, IGFBP3, LAMA2, PRRX2, LAMA3, NEXN, ICAM1, IRS1,	0.0421
i12F/i6F	positive regulation of tyrosine phosphorylation of STAT protein (GO:0042531)	BP	0.98	IL6,CSF2,MAFB	4.36689E-2
i12F/i6F	Positive regulation of gene-specific transcription from RNA polymerase II promoter (GO:0010552)	BP	1.28	HMGB2,NR1H3,E2F1,GDNF,MAFB	0.04461
i12F/i6F	ribonuclease H activity (GO:0004523)	MF	0.77	RNASEH2A, FEN1, EXO1	0.005892
i12F/i6F	chromatin (GO:0000785)	CC	7.18	HIST1H1A,HIST1H3B,HIST1H1B,HIST1H3D,HIST1H1D,HIST1H3F,HIST1	4.12325E-9

				H4D,HIST1H2AL,HIST1H4L,HIST1H2AJ,HIST1H3H,HIST1H2AM,ID2,HIST1H4F,HIST1H4C,HIST2H2AC,HIST1H4J,ASF1B,HIST1H4E,KIF22,POLA1,TIMELESS,E2F1,HIST1H4I,OIP5,ABTB2,VDR,MAFB	
i12F/i6F	nucleosome (GO:0000786)	CC	5.13	HIST1H1A,HIST1H3B,HIST1H1B,HIST1H3D,HIST1H1D,HIST1H3F,HIST1H4D,HIST1H2AL,HIST1H4L,HIST1H2AJ,HIST1H3H,HIST1H2AM,HIST1H4F,HIST1H4C,HIST2H2AC,HIST1H4J,HIST1H4E,HIST1H4I,ABTB2,MAFB	4.12325E-9
i12F/i6F	chromosome, centromeric region (GO:0000775)	CC	3.85	ZWINT,SPAG5,CENPM,C13orf3,BIRC5,KIF2C,MLF1IP,KIF22,CDCA8,ITGB3BP,CENPP,BUB1B,CENPF,OIP5, MAFB	1.1716E-7
i12F/i6F	spindle (GO:0005819)	CC	3.33	SPAG5,CDC2,TPX2,C13orf3,BIRC5,KIF15,KIF22,CDC6,PRC1,BUB1B,PLK1,NUSAP1,MYH9	2.07202E-5
i12F/i6F	spindle microtubule (GO:0005876)	CC	1.54	SPAG5,CDC2,C13orf3,BIRC5,PRC1, NUSAP1	2.31712E-4
i12F/i6F	kinetochore (GO:0000776)	CC	2.05	ZWINT,SPAG5,CENPM,C13orf3,MLF1IP,KIF22,BUB1B,CENPF	9.21283E-4
i12F/i6F	condensed chromosome kinetochore (GO:0000777)	CC	1.54	ZWINT,SPAG5,CENPM,C13orf3,MLF1IP,BUB1B	5.08552E-3
i i12F/i6F	microtubule cytoskeleton (GO:0015630)	CC	5.64	CDC45L,SPAG5,CDC2,TPX2,C13orf3,BIRC5,KIF2C,TOP2A,KIF15,KIF22,CDCA8,CDC6,PRC1,BUB1B,GTSE1,PLK1,CENPF,NUSAP1,CEP55,MYH9,JUB,MAFB	6.25947E-3
i i12F/i6F	condensed chromosome (GO:0000793)	CC	2.31	NCAPG,ZWINT,SPAG5,CENPM,C13orf3,HMGB2,TOP2A,MLF1IP,BUB1B	6.25947E-3
i12F/i6F	integrin complex (GO:0008305)	CC	1.28	ITGA2,MYH9,ITGA11,ITGA1,MAFB	6.56309E-3
i i12F/i6F	condensed chromosome, centromeric region (GO:0000779)	CC	1.54	ZWINT,SPAG5,CENPM,C13orf3,MLF1IP,BUB1B	7.19236E-3
i12F/i6F	secretory granule (GO:0030141)	CC	2.56	PCSK1,GAL,CFD,PLA2G4A,HGF,NPTX1,FSTL3,ITGA1,FN1,MAFB	1.66104E-2
i12F/i6F	Collagen type IV (GO:0005587)	CC	1.25	PRRX2, EGR1, COL4A4,	0.01737
i12F/i6F	Basement membrane (GO:0005604)	CC	2.5	LAMA2, PRRX2, LAMA3, ANXA2P3, EGR1, COL4A4,	0.03728
i12F/i6F	Cell Cycle, Mitotic (REACT_152)	REACTOME	6.15	CDC45L, PKMYT1, PRKAR2B, ZWINT, CENPM, UBE2C, CDC2,TYMS, BIRC5, RRM2, LIG1, KIF2C, MLF1IP, MCM5, CDCA8,ITGB3BP, CDC6, POLA1, CENPP, FEN1, BUB1B, PLK1, E2F1,MAFB,	4.51E-08
i12F/i6F	Telomere Maintenance (REACT_7970)	REACTOME	3.08	HIST1H4D, HIST1H4L, HIST1H2AJ, HIST1H4F, HIST1H4C, LIG1,HIST2H2AC, HIST1H4J, HIST1H4E, POLA1, FEN1, HIST1H4I,	4.51E-08
i12F/i6F	Integrin cell surface interactions (REACT_13552)	REACTOME	2.92	LAMA2, PRRX2, ICAM2, ICAM1, RASGRP2, EGR1, COL4A4,	0.001781
i12F/i6F	DNA Replication (REACT_383)	REACTOME	1.79	CDC45L, LIG1, MCM5, CDC6, POLA1, FEN1, E2F1,	0.03135
i6F	regulation of cell differentiation (GO:0045595)	BP	7.56	HIST1H4B,KRT84,HIST1H4K,IAPP,HIST1H4H,HIST2H4B,CHRNB2,NPPC,SMAD1,SPP1,FGF2,APOE,LIF,ITGB3,JUN,SOD2,HES1	5.55655E-3
i6F	DNA packaging (GO:0006323)	BP	4.89	HIST1H4B,HIST1H4K,HIST1H4H,HIST2H4B,HELLS,CHAF1B,NCAPG2,NCAPD3,HIST1H3G,STRA8,HIST2H2AA4	1.0232E-2
i6F	negative regulation of cell differentiation (GO:0045596)	BP	4.44	HIST1H4B,HIST1H4K,IAPP,HIST1H4H,HIST2H4B,SPP1,LIF,ITGB3,SOD2, HES1	1.45926E-2
i6F	negative regulation of DNA metabolic process	BP	2.22	ENPP7,BLM,IRF1,NPPC,STRA8	1.77042E-2

	(GO:0051053)				
i6F	regulation of developmental process (GO:0050793)	BP	10.22	HIST1H4B,KRT84,HIST1H4K,IAPP,HIST1H4H,HIST2H4B,SNCB,HELLS,CHRNB2,SFRP1,NPPC,SMAD1,SPP1,BIRC3,FGF2,SERPINE1,APOE,LIF,ITGB3,PDPN,JUN,SOD2,HES1	2.83648E-2
i6F	DNA replication (GO:0006260)	BP	4.44	ENPP7,PRIM1,GINS2,CHAF1B,BLM,RFC5,RFC4,IRF1,STRA8,JUN	2.9385E-2
i6F	chromosome (GO:0005694)	CC	7.56	HIST1H4B, HIST1H4K, HIST1H4H, HIST2H4B, HELLS, CENPQ,PRIM1, CENPN, BLM, RFC5, NCAPD3, INCENP, RFC4, HIST1H3G,IRF1, JUN, HIST2H2AA4,	0.02965
i6F	Telomere Maintenance (REACT_7970)	REACTOME	4	HIST1H4B, HIST1H4K, HIST1H4H, HIST2H4B, PRIM1, RFC5, RFC4,IRF1, HIST2H2AA4,	4.71E-07

Supplementary Table S2. Summary of FISH-based ploidy counts in hFSK cells.

*Aneuploid percentages include all the observed aneuploid combinations (1 18: 1 X: 1Y), (2 18: 2 X: 1Y), (2 18: 1 X: 2Y), (3 18: 1 X: 1Y), (3 18: 2 X: 2Y), (3 18: 2 X: 1Y), (2 18: 2 X: 2Y), (2 18: 0 X: 1Y) and (2 18: 1 X: 0Y).

% hFSK NUCLEI	DIPLOID (2 18: 1 X: 1 Y)	HAPLOID (1 18: 1 X: 0 Y)	HAPLOID (1 18: 0 X: 1 Y)	TETRAPLOID (4 18: 2 X: 2 Y)	ANEUPLOID*
MOCK WHOLE CULTURE	100.00 +/- 0.00	0.00 +/- 0.00	0.00 +/- 0.00	0.00 +/- 0.00	0.00 +/- 0.00
MOCK 1N SORTED	99.41 +/- 1.78	0.00 +/- 0.00	0.10 +/- 0.10	0.41 +/- 0.42	0.08 +/- 0.09

i12F WHOLE CULTURE	94.16 +/- 1.64	0.59 +/- 0.26	0.42 +/- 0.18	0.83 +/- 0.53	0.44 +/- 0.26
i12F 1N SORTED	90.38 +/- 1.77	3.07 +/- 1.57	2.61 +/- 1.62	0.66 +/- 0.56	0.36 +/- 0.27
i12F CLUMPS	90.77 +/- 1.85	0.67 +/- 0.33	1.89 +/- 0.21	2,25 +/- 0.91	0,48 +/- 0.29

i6F WHOLE CULTURE	95.40 +/- 1.24	0.58 +/- 0.22	0.48 +/- 0.12	1.00 +/- 1.01	0.27 +/- 0.03
i6F 1N SORTED	84.45 +/- 0.67	8.18 +/- 2.62	4.26 +/- 1.56	0.32 +/- 0.32	0.30 +/- 0.01
i6F CLUMPS	89.03 +/- 2.42	4.91 +/- 3.87	1.04 +/- 0.21	1.35 +/- 0.73	0.40 +/- 0.27

Supplementary Table S3. Summary of FISH-based ploidy counts in hMSC cells.

*Aneuploid percentages include all the observed aneuploid combinations (1 18: 1 X: 1Y), (2 18: 2 X: 1Y), (2 18: 1 X: 2Y), (3 18: 1 X: 1Y), (3 18: 2 X: 2Y), (3 18: 2 X: 1Y), (2 18: 2 X: 2Y), (2 18: 0 X: 1Y) and (2 18: 1 X: 0Y).

% hMSC NUCLEI	DIPLOID (2 18: 1 X: 1 Y)	HAPLOID (1 18: 1 X: 0 Y)	HAPLOID (1 18: 0 X: 1 Y)	TETRAPLOID (4 18: 2 X: 2 Y)	ANEUPLOID*
MOCK WHOLE CULTURE	100.00 +/- 0,00	0.00 +/- 0.00	0.00 +/- 0.00	0.00 +/- 0.00	0.00 +/- 0.00
MOCK 1N SORTED	87.66 +/- 4.96	0.00 +/- 0.00	0.00 +/- 0.00	2.32 +/- 2.33	1.11 +/- 0.50
i12F WHOLE CULTURE	91.82 +/- 2.05	0.75 +/- 0.19	0.24 +/- 0.18	2.46 +/- 0.89	0.52 +/- 0.38
i12F 1N SORTED	76.84 +/- 4.39	17.79 +/- 6.26	0.91 +/- 0.70	0.00 +/- 0.00	0.49 +/- 0.30
i6F WHOLE CULTURE	90.90 +/- 2.15	2.28 +/- 0.74	1.82 +/- 0.70	3.93 +/- 1.27	0.11 +/- 0.08
i6F 1N SORTED	80.00 +/- 2.35	20.00 +/- 0.98	0.00 +/- 0.00	0.00 +/- 0.00	0.00 +/- 0.00

Supplementary Table S4. Primer sequences 5´-3' used to gene expression analysis by RT-qPCR.

GENE	PRIMER SEQUENCES	LENGTH
**PRDM1**	F: GCCAAGTTCACCCAGTTTGT	183
	R: GATTCGGGTCAGATCTTCCA	
**PRDM14**	F: TCTCTACGATCTGCCCTGGT	231
	R: CTCAGCCCCTCAGGTAACAG	
**LIN28A**	F: CTTCTCCGAACCAACCCTTT	239
	R: CGCACGTTGAACCACTTACA	
**NANOG**	F: GATTTGTGGGCCTGAAGAAA	155
	R: AAGTGGGTTGTTTGCCTTTG	
**NANOS3**	F: GGGAAAGAGGGTCCTGAAAC	221
	R: AGCACGTGGGACTGGTAGAT	
**DAZ2**	F: GAGATTGGAAGCTGCTTTGG	212
	R: TGCACATGACGAGCACATAA	
**DAZL**	F: AATGACGTGGATGTGCAGAA	152
	R: AACTGTGGTGGAGGAGGATG	
**BOLL**	F: CTAATCCTGTGTCACCTGTGC	205
	R: ACGAAACCATACCCTTTGGA	
**VASA**	F: ATGGATGATGGACCTTCTCGA	248
	R: CCTCTGTTCCGTGTTGGATT	
**STRA8**	F: AATCCCATGACAGAGCAAC	227
	R: TTATCCAGGGTTTGCTCCAG	
**SYCP3**	F: AGCCGTCTGTGGAAGATCAG	197
	R: AACTCCAACTCCTTCCAGCA	
**DMC1**	F: CTTTCAGGCAGATCCCAAAA	172
	R: CCCAATTCCTCCAGCAGTTA	
**CDH1**	F: TGAAGGTGACAGAGCCTCTGGAT	151

	R: TGGGTGAATTCGGGCTTGTT	
**STELLA**	F: CAGTCTCAGGGAAATCGAA	203
	R: GCAGAAACTGCAGGGACATT	
**FRAGILIS**	F: ATGTCGTCTGGTCCCTGTTC	267
	R: AACCCCGTTTTTCCTGTATT	
**GFRA1**	F: GCAAGGAGACCAACTTCAGC	190
	R: TCCTCCAGCAGATGATTTCC	
**PIWIL2**	F: GTTAATGGTGATCGGGATGG	120
	R: ATGCATGCCATTTATCAGCA	
**TNP2**	F: CCAACACTAGTCCACCACCA	197
	R: GTTGGATTTCCATCCTGAGC	
**PRM1**	F: CCGCCAGAGACAAAGAAGTC	200
	R: GGATGGTGGCATTTTCAAGA	
**ACRO**	F: ATTCTGCTGGTCTTGGCAGT	192
	R: TGTGTGGTACCTGTGGCTGT	
**DNMT1**	F: TACCTGGACGACCCTGACCTC	103
	R: CGTTGGCATCAAAGATGGACA	
**DNMT3A**	F: TATTGATGAGCGCACAAGAGAGC	111
	R: GGGTGTTCCAGGGTAACATTGAG	
**DNMT3B**	F: GGCAAGTTCTCCGAGGTCTCTG	188
	R: TGGTACATGGCTTTTCGATAGGA	
**TET1**	F: GCTATACACAGAGCTCACAG	139
	R: GCCAAAAGAGAATGAAGCTCC	
**TET2**	F: CTTTCCTCCCTGGAGAACAGCTC	146
	R: TGCTGGGACTGCTGCATGACT	
**TET3**	F: ATTCTGCTGGTCTTGGCAGT	193
	R: TGTGTGGTACCTGTGGCTGT	
**RPL19**	F: GTTCCTGGAGCATGTACTTC	199
	R: CTTCCTCTTTGGGATTGTCC	

**T (BRACHYURY)**	F: TCAGCAAAGTCAAGCTCACCA<br>R: CCCCAACTCTCACTATGTGGATT	102
**SOX17**	F: TCTGCCTCCTCCACGAAG<br>R: CAGAATCCAGACCTGCACAA	101
**CD38**	F: TTGGGAACTCAGACCGTACC<br>R: GTTGCTGCAGTCCTTTCTCC	200
**TFCP2L1**	F: GTGTACCACGCCATCTTCCT<br>R: TGTGCTGAGGACAAAACAGG	176
**FIGLα**	F: AATCTCAACCGTGGTTTTGC<br>R: CTTGCCGAGGATCTATGTGA	215
**GDF9**	F: GGGAGAAGCTCAGATTGCTG<br>R: GGAATCCCTTCCTTGGTAGC	225
**ZP1**	F: CGCCATGTTCTCTGTCTCAA<br>R: CGTTTGTTCACATCCCAGTG	161

Supplementary Table S5. List of the antibodies employed.

ANTIGEN	HOST	COMPANY	DILUTION
**VIMENTIN**	Mouse	Dako	1:200
**Human PLZF**	Mouse	R&D	1:50
**UTF1**	Mouse	Milipore	1:50
**VASA**	Goat	R&D	1:200
**DAZL**	Rabbit	Abcam	1:200
**HIWI**	Rabbit	Novex	1:200
**Human-SYCP3**	Rabbit	Novus	1:75
**Human-centromere protein (CREST)**	Human	Fisher	1:1000
**5-methyl Cytosine (5-mC)**	Mouse	Eurogentec	1:500
**5-hydroxymethyl Cytosine (hmC)**	Rabbit	Active motif	1:200
**Human NuMA**	Rabbit	Abcam	1:200
**3ß-HSD**	Mouse	Abcam	1:50
**FSHR**	Rabbit	Abcam	1:50
**SOX9**	Mouse	Abcam	1:50
